# An integrated mathematical model for optimizing integrated pest management strategies against rice tungro virus disease

**DOI:** 10.1038/s41598-025-22236-3

**Published:** 2025-11-10

**Authors:** Rika Amelia, Nursanti Anggriani

**Affiliations:** 1 Post Doctoral Program, Department of Mathematics, Faculty of Mathematics and Natural Science, Universitas Padjadjaran, Bandung, Indonesia; 2 Department of Mathematics, Faculty of Mathematics and Natural Sciences, Universitas Kebangsaan Republik Indonesia, Bandung, Indonesia; 3 Department of Mathematics, Faculty of Mathematics and Natural Science, Universitas Padjadjaran, Bandung, Indonesia

**Keywords:** Optimal Control, Tungro, Roguing, Insecticides, Natural Enemies, Mathematics and computing, Applied mathematics

## Abstract

One of the main obstacles in rice cultivation is rice tungro disease, caused by the combined infection of *Rice Tungro Spherical Virus* (RTBV) and *Rice Tungro Spherical Virus* (RTSV), which are transmitted by green leafhopper vectors (*Nephotettix virescens*) through a semi-persistent mode of transmission. Control of this disease can be carried out using roguing, insecticide application, and the planting of refugia plants. Each control strategy has its advantages and disadvantages. Thus, analysis is needed to determine the most effective approach. Mathematically, one way to evaluate the effectiveness of these control methods is by developing a mathematical model of the spread of rice tungro disease that considers the characteristics of the viruses, the presence of vector and natural enemies, roguing, refugia planting, and insecticide use. Dynamic and sensitivity analyses, along with optimal control strategies, were conducted based on the model. The results indicate that the non-endemic equilibrium point is locally asymptotically stable if $R_0<1$, with key influencing parameters being the insecticide-induced mortality rate $(\mu _2)$ and the natural enemy recruitment rate $(\xi )$. Numerical simulations suggest that combining roguing, refugia planting, and insecticide application while utilizing natural enemies is the most efficient control strategy.

## Introduction

Rice tungro disease is a serious problem that can lead to significant crop yield losses and negatively impact the economy, particularly in Indonesia. This disease results from a dual infection by the *Rice Tungro Bacilliform Virus* (RTBV) and the *Rice Tungro Spherical Virus* (RTSV). It is not transmitted through seeds or mechanical means but instead requires a vector insect, namely the green leafhopper (*Nephotettix virescens*), which transmits the virus in a semipersistent manner. Transmission occurs when the vector feeds on an infected plant and subsequently moves to a healthy one without undergoing a latent period within its body. While the green leafhopper can transmit RTSV independently, it cannot transmit RTBV without the presence of RTSV. RTBV is considered the primary agent of damage as it induces severe symptoms such as stunting, leaf discoloration, and significant yield reduction. RTSV, while capable of independent transmission, does not cause substantial symptoms on its own but facilitates RTBV transmission by enabling the vector to transmit it more efficiently. The synergistic interaction between RTBV and RTSV significantly amplifies the severity and spread of the disease^1–5^.

Because the green leafhopper plays a crucial role in the spread of tungro disease, it is essential to implement control strategies to suppress its population and reduce disease transmission in rice plants^6,7^. Several control methods are available, including roguing (removal of infected plants), pesticide or insecticide application, and the use of natural enemies. Each method has its advantages and drawbacks. Roguing can improve crop quality but may reduce yields if performed continuously. Pesticides and insecticides provide a faster approach to controlling the spread of disease. However, excessive use can lead to pest and disease resistance, environmental harm, and the destruction of beneficial organisms. On the other hand, the use of natural enemies is more environmentally friendly and cost-effective. This method can be supported by planting refugia plants to attract and sustain populations of natural enemies. Nevertheless, this biological control strategy tends to be slower in showing results compared to chemical methods. Therefore, to better assess and optimize the effectiveness of roguing, pesticide and insecticide use, and natural enemy deployment (including refugia planting), mathematical modeling becomes a valuable tool^8^.

Numerous researchers have developed mathematical models to study the spread of plant diseases. Some models combine roguing and replanting as prevention strategies, as studied by Anggriani^9,10^. In the following year, Anggriani considered curative and preventive controls, as well as multiple infection events^11,12^. In 2020, Anggriani further developed the model by incorporating optimal control strategies for roguing, replanting, and chemical or biological interventions^13^. Another model, developed by Anggriani and Amelia^11,14^, explored the use of botanical fungicides to reduce disease intensity. Mathematical models have also been applied to other plant diseases, such as fungal infections. Anggriani et al.^9^ developed such models, later enhanced to incorporate predator-based controls^15^. In another example, Amelia et al.^16^ modeled the spread of yellow disease in chili plants. These models were further refined by incorporating optimal control strategies using *Verticillium lecanii* and logistic growth functions for vector populations^17,18^.

Research on the rice tungro virus disease has also made significant progress. Anggriani et al.^9^ developed a model considering insecticide use, later extended to include biological agents^15^. In 2022, Suryaningrat et al.^19^ introduced spatial environmental aspects into the model. Meanwhile, Maryati et al.^20^ analyzed the transmission of tungro disease using matrix methods, incorporating the growth phases of rice (vegetative and generative). Their model was then expanded to include the use of pesticide suicides and natural enemies^6^. Blas et al.^21^ developed a model that considered the transmission characteristics of viruses, which was further refined in subsequent work^22^. Based on this, Amelia et al.^6^ incorporated pesticide application into the model and performed dynamic analysis. The model was later extended to include roguing^7^.

Since many parameter values in mathematical modeling are assumed due to limited data availability, sensitivity analysis is crucial for identifying parameters that significantly influence the model’s behavior^23–28^. In addition, optimal control theory has been widely applied in mathematical modeling to determine the most effective and cost-efficient disease management strategies. In plant disease epidemiology, this approach enables the quantitative evaluation of combined interventions, such as roguing, insecticide application, and biological control using natural enemies. For instance, Anggriani et al.^13^ applied optimal control to disease spread models incorporating multiple interventions. Amelia et al.^17,18^ also developed optimal control models for chili yellow disease, including the dynamics of natural enemies such as *Verticillium lecanii*. Foundational works by Fleming and Rishel^29^ provide the theoretical basis for deterministic and stochastic optimal control in biological systems, while Schroers^30^ discusses numerical methods for optimal control applications in environmental management. Other studies, such as Nundloll et al.^31^ and O’Regan & Kyrychko^32^, demonstrate the application of optimal control in pest and disease management with resource constraints. Moreover, recent research in a related but different field, such as the study by Hye et al. (2025)^33^ on the co-infection of COVID-19 and kidney disease, highlights how optimal control can be used to reduce health complexity through integrated interventions.

Collectively, these works highlight the potential of optimal control theory to develop effective and economically viable disease management strategies in agricultural systems. Based on these insights, this study develops a new mathematical model for rice tungro disease transmission that integrates virus transmission characteristics, roguing, biological control using natural enemies, refugia planting, and insecticide application. The model is analyzed dynamically, followed by sensitivity analysis to identify critical parameters. Furthermore, optimal control theory is applied to determine the most cost-effective combination of interventions. The results are expected to enhance the understanding of disease dynamics and inform practical, data-driven strategies for managing tungro disease in rice fields.

## Models

The rice tungro disease model was developed based on the framework proposed by Amelia et al.^6^, which integrates various control strategies, including roguing, insecticide application, and biological control through the planting of refugia, to support sustainable disease management. This model adopts an approach that accounts for the characteristics of the two tungro viruses, RTSV and RTBV, as described in previous studies^6,21,34^. Natural enemy conservation strategies, such as the planting of refugia plants, have proven to be effective in suppressing green leafhopper vector populations while simultaneously improving the abundance of natural enemies, without negatively impacting crop yields. This approach is consistent with the principles of Integrated Pest Management (IPM), which emphasizes minimizing the use of pesticides.

The mathematical model divides the populations into three main groups: rice plants, green leafhopper vectors, and natural enemies. Rice plants are classified into four compartments: susceptible ($P_0$), RTSV-infected ($P_1$), RTBV-infected ($P_2$), and co-infected with both viruses ($P_3$). Vectors follow a similar classification, while natural enemies are not subdivided into classes^6^. Susceptible plants remain in this category unless exposed to a viruliferous vector. Since tungro viruses are transmitted semi-persistently without a latent period, infected plants are assumed to be irrecoverable, leading to permanent loss of the susceptible population^6,21^.

In addition, the model incorporates population growth functions for both vectors and rice plants. For vector growth ($V_0$), the function $BN_v(1 - \frac{N_v}{K})$ represents logistic growth, where *B* is the per capita growth rate and *K* is the maximum environmental carrying capacity. This assumes that the vector population $N_v$ increases over time, but is ultimately constrained by the habitat capacity *K*. For the growth of rice plants ($P_0$), the function $r(K - N_p)$ is used, where *r* denotes the intrinsic growth rate of rice plants and $(K-N_p)$ represents the remaining environmental capacity available to the plant population $N_p$. Unlike the logistic form, this function is expressed linearly, with the assumption that plant growth is directly proportional to the availability of unallocated space or resources.

Vector infection dynamics reflect the interaction with infected plants: vectors may acquire RTSV first and then RTBV, or both viruses simultaneously from a co-infected plant^6^. Vectors feeding on RTSV-infected plants can transmit the virus to susceptible plants, whereas vectors feeding exclusively on RTBV-infected plants are unable to transmit it. Transmission of both viruses occurs when vectors feed on plants infected with RTSV, followed by plants infected with RTBV, or from plants carrying both viruses. After the retention period, vectors initially infected with both viruses eventually retain only RTBV, while those infected solely with RTBV may revert to a susceptible state^6,34^.

Vector population control within the model encompasses both biological and chemical approaches, including the conservation of natural enemies through the establishment of refugia and the application of insecticides as needed. Refugia provide habitats that improve the populations of natural enemies, thereby suppressing vector abundance without overreliance on pesticides. Mortality among vectors may result from predation by natural enemies or insecticide application, while natural enemies may experience natural mortality or pesticide-induced mortality^6^.

**Fig. 1:**
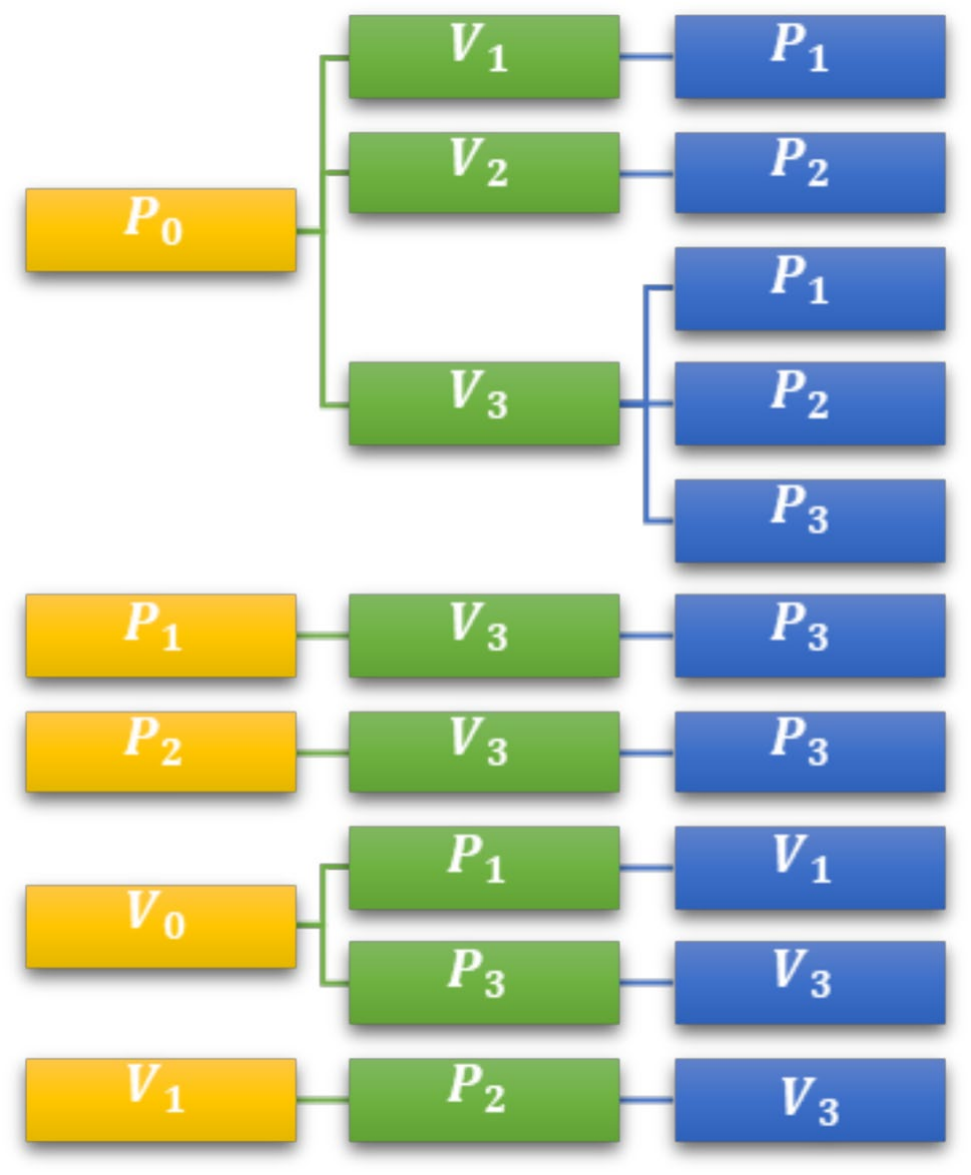
Infection transition diagram.

**Fig. 2:**
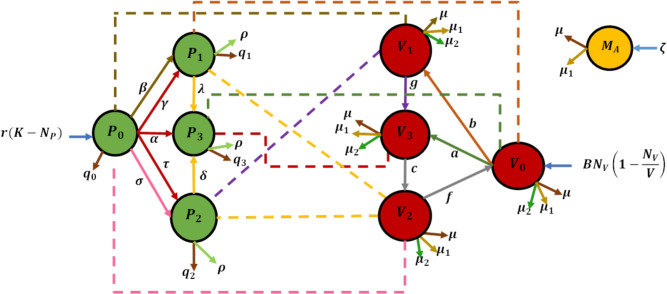
Schematic diagram.

Figs. 1 and 2 illustrate interactions and transitions between vectors and plants under the assumption that virus-free vectors become infectious after feeding twice on infected plants. The definitions of parameters are provided in Table 1. This integrated modeling approach reveals how biological control can reduce reliance on insecticides while efficiently restricting infection sources, together achieving greater disease suppression than any single control method alone.

**Table 1:** Definition of parameters and variables.

Parameter	Description	Value
$\alpha$	Transition rate $P_0 \rightarrow P_3$ due to transition $V_3$	0,035 $\frac{Plant}{Vector \times day}$^21,22^
$\beta$	Transition rate $P_0 \rightarrow P_1$ due to transition $V_1$	0,09 $\frac{Plant}{Vector \times day}$^21,22^
$\gamma$	Transition rate $P_0 \rightarrow P_1$ due to transition $V_3$	0,01 $\frac{Plant}{Vector \times day}$^21,22^
$\sigma$	Transition rate $P_0 \rightarrow P_2$ due to transition $V_2$	0,08 $\frac{Plant}{Vector \times day}$^21,22^
$\tau$	Transition rate $P_0 \rightarrow P_2$ due to transition $V_2$	0,06 $\frac{Plant}{Vector \times day}$^21,22^
$\delta$	Transition rate $P_2 \rightarrow P_3$ due to transition $V_3$	0,07 $\frac{Plant}{Vector \times day}$^21,22^
*a*	Transition rate $V_0 \rightarrow V_3$ due to acquisition of $P_3$	0,996 $\frac{Plant}{Vector \times day}$^21,22^
*b*	Transition rate $V_0 \rightarrow V_1$ due to acquisition of $P_1$	0,996 $\frac{Plant}{Vector \times day}$^21,22^
$q_0$	Natural death of susceptible plants	0,008 $\frac{1}{day}$^21,22^
$q_1$	Natural death of RTSV plants	0,009 $\frac{1}{day}$^21,22^
$q_2$	Natural death of RTBV plants	0,0125 $\frac{1}{day}$^21,22^
$q_3$	Natural death of RTSV+RTBV plants	0,0125 $\frac{1}{day}$^21,22^
*r*	Plant recruitment rate	0,001 $\frac{1}{day}$^21,22^
*B*	Vector recruitment rate	0,033 $\frac{1}{day}$^21,22^
*V*	Maximum vector capacity	100.000 Vector^21,22^
*K*	Maximum plant capacity	30.000 Plant^21,22^
$\lambda$	Transition rate $P_1 \rightarrow P_3$ due to transition $V_3$	0,03 $\frac{Plant}{Vector \times day}$^21,22^
$\mu$	Natural death	0,033 $\frac{Plant}{Vector \times day}$^21,22^
$\mu _1$	Deaths due to insecticides	0,033 $\frac{Plant}{Vector \times day}$^21,22^
$\mu _2$	Death due to natural enemies	0,033 $\frac{Plant}{Vector \times day}$^21,22^
*c*	Retention period of RTSV$+$RTBV vector	0,5 $\frac{Plant}{Vector \times day}$^21,22^
*f*	Retention period of RTSV$+$RTBV vector	0,33 $\frac{Plant}{Vector \times day}$^21,22^
*g*	Transition rate $V_1 \rightarrow V_3$ due to acquisition of $P_2$	0,996 $\frac{Plant}{Vector \times day}$^21,22^
$\rho$	Death rate due to roguing	0,0001 $\frac{1}{day}$^7^
$\xi$	Recruitment natural enemies	0,01 $\frac{1}{day}$^6^

From the intention transition and schematic diagram in Fig. 2 and the definition of parameters and variables in Table 1, the model formed is as follows:

1 $$\begin{aligned} \frac{dP_0}{dt}&= r(K-N_P)-\frac{\alpha P_{0}V_{3}}{N_{P}}-\frac{\gamma P_{0}V_{3}}{N_{P}}-\frac{\tau P_{0}V_{3}}{N_{P}}-\frac{\beta P_{0}V_{1}}{N_{P}}-\frac{\sigma P_{0}V_{2}}{N_{P}}-q_{0}P_{0} \end{aligned}$$

2 $$\begin{aligned} \frac{dP_{1}}{dt}&= \frac{\beta P_{0} V_{1}}{N_{P}}+\frac{\gamma P_{0} V_{1}}{N_{P}}-\frac{\lambda P_{1} V_{3}}{N_{P}}-q_{1} P_{1}-\rho P_{1}\end{aligned}$$

3 $$\begin{aligned} \frac{dP_{2}}{dt}&= \frac{\tau P_{0} V_{3}}{N_{P}}+\frac{\sigma P_{0} V_{2}}{N_{P}}-\frac{\delta P_{2} V_{3}}{N_{P}}-q_{2} P_{2}-\rho P_{2}\end{aligned}$$

4 $$\begin{aligned} \frac{dP_{3}}{dt}&= \frac{\alpha P_{0} V_{3}}{N_{P}}+\frac{\lambda P_{0} V_{3}}{N_{P}}-\frac{\delta P_{2} V_{3}}{N_{P}}-q_{3} P_{3}-\rho P_{3}\end{aligned}$$

5 $$\begin{aligned} \frac{dV_{0}}{dt}&= BN_{V}\bigg (1-\frac{N_{V}}{V}\bigg )-\frac{a V_{0} P_{3}}{N_{P}}+\frac{b V_{0} P_{1}}{N_{P}}+f V_{2}-(\mu _{2} M_{A}+\mu _{1}+\mu ) V_{0}\end{aligned}$$

6 $$\begin{aligned} \frac{dV_1}{dt}&= \frac{bV_{0}P_{1}}{N_P}-\frac{gV_{1}P_{2}}{N_P}-(\mu _{2} M_{A}+\mu _{1}+\mu ) V_{1}\end{aligned}$$

7 $$\begin{aligned} \frac{dV_2}{dt}&=-fV_2+cV_3-(\mu _{2} M_{A}+\mu _{1}+\mu ) V_{2}\end{aligned}$$

8 $$\begin{aligned} \frac{dV_3}{dt}&= \frac{V_{0}P_{3}}{N_P}-\frac{gV_{1}P_{2}}{N_P}-cV_3-(\mu _{2} M_{A}+\mu _{1}+\mu ) V_{3}\end{aligned}$$

9 $$\begin{aligned} \frac{dM_A}{dt}&= \phi \mu _{2} M_{A} N_{V}+\xi M_{A}-(\mu +\mu _1)M_A \end{aligned}$$

with $$P_i(0),V_i(0),M_A(0)\ge 0;i=0, 1, 2, 3.$$

Equations (1)–(9) represent the dynamics of tungro disease transmission in rice plants and its vector, incorporating three control strategies: roguing, insecticide application, and biological control. Equation (1) describes the dynamics of susceptible plants ($P_0$), which increase through replanting at a rate of $r(K-N_P)$ and decrease due to infection by vectors ($V_1$, $V_2$, and $V_3$) as well as the removal of healthy plants through roguing. Equations (2) and (3) describe the transition of plants to singly infected states with RTSV ($P_1$) and RTBV ($P_2$), respectively, through interactions with infected vectors. These can further progress to doubly infected plants ($P_3$), as described in Equation (4). Each infected plant class may decrease due to roguing or natural loss (harvesting or death). Meanwhile, Equations (5)–(8) model the vector dynamics across four classes: susceptible vectors ($V_0$) increase through logistic recruitment and decrease due to infection from infected plants, as well as death from insecticides, natural enemies ($M_A$), and natural mortality. Vectors can transition between infection classes (RTSV, RTBV, or co-infection) through interaction with infected plants. Equation (9) describes the growth of natural enemies, which increases through the utilization of vectors as hosts and natural reproduction and decreases due to natural mortality and exposure to insecticides.

## Dynamic analysis

### Existence, uniqueness, non-negativity, and bounded of solutions

#### Theorem 1

The solution obtained from the mathematical model of the spread of tungro disease in rice plant equations (1) to (9) has a unique solution,$H(t)=(P_0(t),P_1(t),P_(t),P_3(t),V_0(t),V_1(t),V_2(t),V_3(t),M_A(t))^T \in \Omega _L$ for $t>0.$ If it has nonnegative initial conditions $(P_i(0),V_i(0),M_A(0)\ge 0;i=0,1,2,3)$.

#### Proof

Suppose

$$\begin{aligned}F(H)=(F_1 (H),F_2 (H),F_3 (H),F_4 (H),F_5 (H),F_6 (H),F_7 (H),F_8 (H),F_9 (H))^T, \end{aligned}$$

where:

$$\begin{aligned}&F_1 (H)=\frac{dP_0}{dt},F_2 (H)=\frac{dP_1}{dt},F_3 (H)=\frac{dP_2}{dt},F_4 (H)=\frac{dP_3}{dt},F_5 (H)=\frac{dV_0}{dt},F_6 (H)=\frac{dV_1}{dt},F_7 (H)=\frac{dV_2}{dt},F_8 (H)=\frac{dV_3}{dt},\\&F_9 (H)=\frac{dM_A}{dt}.\end{aligned}$$

For any $H, H^*\in \Omega _L$ obtained:

$$\begin{aligned} \Vert F(H) - F(H^*)\Vert&\le M \big | (P_0 - P_0^*) + (P_1 - P_1^*) + (P_2 - P_2^*) + (P_3 - P_3^*) \\&\quad + (V_0 - V_0^*) + (V_1 - V_1^*) + (V_2 - V_2^*) + (V_3 - V_3^*) + (M_A - M_A^*) \big | \end{aligned}$$

Let $$M = \max \{M_1, M_2, M_3, M_4, M_5, M_6, M_7, M_8, M_9\}$$.

It is clear that *F*(*H*) satisfies the Lipschitz condition on *H*. Therefore, the system of equations (1)–(9) with nonnegative initial conditions

$$P_i(0) \ge 0,\quad V_i(0) \ge 0,\quad M_A(0) \ge 0,\quad \text {for } i = 0,1,2,3,$$

has a unique solution. $\square$

#### Theorem 2

If all initial conditions satisfy $P_i(0) \ge 0$, $V_i(0) \ge 0$, and $M_A(0) \ge 0$ for $i = 0,1,2,3$, then the solutions of equations (1)–(9) remain non-negative for all $t> 0$.

#### Proof

equations (1) to (9) are positive for $t>0$. Assuming the initial conditions are nonnegative, $P_0(0)\ge 0, P_1(0)\ge 0, P_2(0)\ge 0, P_3(0)\ge 0, V_0(0)\ge 0, V_1(0)\ge 0, V_2(0)\ge 0, V_3(0)\ge 0, M_A(0)\ge 0$, we get:

10 $$\begin{aligned} \frac{dP_0}{dt}&= r(K - N_P) - \frac{\alpha P_0 V_3}{N_P} - \frac{\gamma P_0 V_3}{N_P} - \frac{\tau P_0 V_3}{N_P} - \frac{\beta P_0 V_1}{N_P} - \frac{\sigma P_0 V_2}{N_P} - q_0 P_0 \nonumber \\ P_0(t)&= P_0(0) \exp \left( -\int \left( \frac{\alpha V_3}{N_P} - \frac{\gamma V_3}{N_P} - \frac{\tau V_3}{N_P} - \frac{\beta V_1}{N_P} - \frac{\sigma V_2}{N_P} - q_0 \right) \, dt \right) \ge 0 \end{aligned}$$

11 $$\begin{aligned} \frac{dP_1}{dt}&= \frac{\beta P_0 V_1}{N_P} + \frac{\gamma P_0 V_3}{N_P} - \frac{\lambda P_1 V_3}{N_P} - q_1 P_1 - \rho P_1 \nonumber \\ P_1(t)&= P_1(0) \exp \left( -\int \left( \frac{\lambda V_3}{N_P} + q_1 + \rho \right) dt \right) \ge 0 \end{aligned}$$

12 $$\begin{aligned} \frac{dP_2}{dt}&= \frac{\tau P_0 V_3}{N_P} + \frac{\sigma P_0 V_2}{N_P} - \frac{\delta P_2 V_3}{N_P} - q_2 P_2 - \rho P_2 \nonumber \\ P_2(t)&= P_2(0) \exp \left( -\int \left( \frac{\delta V_3}{N_P} + q_2 + \rho \right) dt \right) \ge 0 \end{aligned}$$

13 $$\begin{aligned} \frac{dP_3}{dt}&= \frac{\alpha P_0 V_3}{N_P} + \frac{\lambda P_1 V_3}{N_P} + \frac{\delta P_2 V_3}{N_P} - q_3 P_3 - \rho P_3 \nonumber \\ P_3(t)&= P_3(0) \exp \left( -\int (q_3 + \rho ) dt \right) \ge 0 \end{aligned}$$

14 $$\begin{aligned} \frac{dV_0}{dt}&= B N_V \left( 1 - \frac{N_V}{V}\right) - \frac{a V_0 P_3}{N_P} - \frac{b V_0 P_1}{N_P} + f V_2 - (\mu _2 M_A + \mu _1 + \mu ) V_0 \nonumber \\ V_0(t)&= V_0(0) \exp \left( -\int \left( \frac{a P_3}{N_P} + \frac{b P_1}{N_P} + \mu + \mu _1 + \mu _2 M_A \right) dt \right) \ge 0 \end{aligned}$$

15 $$\begin{aligned} \frac{dV_1}{dt}&= \frac{b V_0 P_1}{N_P} - \frac{g V_1 P_2}{N_P} - (\mu _2 M_A + \mu _1 + \mu ) V_1 \nonumber \\ V_1(t)&= V_1(0)\exp \left( -\int \left( \frac{g P_2}{N_P} + \mu + \mu _1 + \mu _2 M_A \right) dt \right) \ge 0 \end{aligned}$$

16 $$\begin{aligned} \frac{dV_2}{dt}&= -f V_2 + c V_3 - (\mu _2 M_A + \mu _1 + \mu ) V_2 \nonumber \\ V_2(t)&= V_2(0)\exp \left( -\int \left( f + \mu + \mu _1 + \mu _2 M_A \right) dt \right) \ge 0 \end{aligned}$$

17 $$\begin{aligned} \frac{dV_3}{dt}&= \frac{a V_0 P_3}{N_P} + \frac{g V_1 P_2}{N_P} - c V_3 - (\mu _2 M_A + \mu _1 + \mu ) V_3 \nonumber \\ V_3(t)&= V_3(0)\exp \left( -\int \left( c + \mu + \mu _1 + \mu _2 M_A \right) dt \right) \ge 0 \end{aligned}$$

18 $$\begin{aligned} \frac{dM_A}{dt}&= \varphi \mu _2 M_A N_V + \xi M_A - (\mu + \mu _1) M_A \nonumber \\ M_A(t)&= M_A(0)\exp \left( -\int (\mu + \mu _1) dt \right) \ge 0 \end{aligned}$$

From equations(10) to (18) we obtain: $P_1,P_2,P_3,V_0,V_1,V_2,V_3,M_A\ge 0$. So it is proven that the solution of equations (1) to (9) is non-negative $(P_0(t)\ge 0,P_1(t)\ge 0,P_2(t)\ge 0,P_3(t)\ge 0,V_0(t)\ge 0,V_1(t)\ge 0,V_2(t)\ge 0,V_3(t)\ge 0,M_A(t)\ge 0)$ for every $t>0$. $\square$

#### Theorem 3

If equations (1) to (9) have solutions, then the obtained solutions are bounded for all $t \in [0, t_0]$.

#### Proof

Suppose $N = N_P + N_V + M_A, \quad N_P = P_0 + P_1 + P_2 + P_3, \quad N_V = V_0 + V_1 + V_2 + V_3,$ with $q_0 = q_1 = q_2 = q_3 = \mu + \mu _1 = \rho = q, \quad \Lambda = r(K - N_P), \quad \omega = B N_V \left( 1 - \frac{N_V}{V} \right) ,$ and $\zeta = \xi M_A.$

From equations (1) to (9), we obtain:

$$\frac{dN}{dt} = \Lambda + \omega + \zeta - qN$$

Using the variable separator, we obtain:

$$\int \frac{dN}{\Lambda + \omega + \zeta - qN} = \int dt.$$

Let $$u = \Lambda + \omega + \zeta - qN \quad \Longleftrightarrow \quad du = -q\,dN.$$

So, $$\lim _{t \rightarrow \infty } N(t) = \lim _{t \rightarrow \infty } \left( \frac{\Lambda + \omega + \zeta }{q} - e^{-(t + C)} \right) = \frac{\Lambda + \omega + \zeta }{q}.$$

Since $N(t) = \frac{\Lambda + \omega + \zeta }{q} - e^{-(t + C)}$ is a monotonically increasing function and $\lim _{t \rightarrow \infty } N(t) = \frac{\Lambda + \omega + \zeta }{q},$ it follows that $0 \le N(t) \le \frac{\Lambda + \omega + \zeta }{q}.$ Therefore, it is proven that equations (1) to (9) are bounded for all $t \in [0, t_0]$. $\square$

### Equilibrium point

The mathematical model of the spread of tungro virus disease in rice plants obtained four equilibrium points: three non-endemic and one endemic equilibrium point.

#### Non-endemic equilibrium point

The non-endemic equilibrium point is obtained by making the infected compartment equal to zero (equations (2) to (4) and (6) to (8) are equal to zero) so that three non-endemic equilibrium points are obtained, namely:

- There are only susceptible rice plants (Case 1):
  19 $$\begin{aligned} E_0=\{P_0,P_1,P_2,P_3,V_0,V_1,V_2,V_3,M_A)=\{\frac{rK}{r+q_0 },0,0,0,0,0,0,0,0\} \end{aligned}$$
- There are only rice plants and susceptible vectors (Case 2)
  20 $$\begin{aligned} E_1=\{P_0,P_1,P_2,P_3,V_0,V_1,V_2,V_3,M_A)\}=\{\frac{rK}{r+q_0},0,0,0,\frac{V(B-(\mu +\mu _1))}{B},0,0,0,0\} \end{aligned}$$
- There are rice plants and susceptible vectors, and natural enemies are present (Case 3)
  21 $$\begin{aligned} E_2&=\{P_0,P_1,P_2,P_3,V_0,V_1,V_2,V_3,M_A\} \nonumber \\&=\{\frac{rK}{r+q_0},0,0,0,\frac{\mu +\mu _1-\xi }{\mu _2 \phi },0,0,0,\frac{(B-(\mu +\mu _1))\mu _2V\phi -B(\mu +\mu _1-\xi )}{\mu _2^2 V \phi }\} \end{aligned}$$

#### Endemic equilibrium point

The endemic equilibrium point is obtained by setting equations (1) to (9) equal to zero, resulting in the following:

22 $$\begin{aligned} E_3 = \{P_0, P_1, P_2, P_3, V_0, V_1, V_2, V_3, M_A\} = \{P_0^*, P_1^*, P_2^*, P_3^*, V_0^*, V_1^*, V_2^*, V_3^*, M_A^*\}. \end{aligned}$$

Since finding the endemic equilibrium point analytically is too complex, a numerical approach is employed to demonstrate its existence. Based on the parameter values listed in Table1, the equilibrium point is computed as follows:

$$\begin{aligned} E_3 = \{P_0^*, P_1^*, P_2^*, P_3^*, V_0^*, V_1^*, V_2^*, V_3^*, M_A^*\}=\{36, 16, 37, 33, 6224, 156, 1011, 1276, 38\}. \end{aligned}$$

### Basic reproduction number

The basic reproduction number $(R_0)$ is the ability of new infections to spread. $R_0$ for the spread of rice tungro disease, considering the presence of vectors and natural enemies in this model, is determined using the next-generation matrix method formulated by vandenDriessche and Watmough^35^. Thus obtained:

23 $$\begin{aligned} R_{01}=\pm \sqrt{\frac{Vb\beta (r+q_0)(\xi -(\mu +\mu _1))}{KrB(\mu +\mu _1-(\xi +\mu _2V\phi ))(q_1+\rho )}}\end{aligned}$$

24 $$\begin{aligned} R_{02}=\pm \sqrt{\frac{Va\alpha (r+q_0)(\xi -(\mu +\mu _1))}{Kr(B(\mu +\mu _1-\xi )-(B+c)\mu _2V\phi )(q_3+\rho )}} \end{aligned}$$

with:

- $R_{01}$:Basic reproduction number for RTSV
- $R_{02}$:Basic reproduction number for RTSV+RTBV
- $$R_0=\max \{R_{01},R_{02}\}.$$

### Stability analysis

#### Theorem 4

The local stability of the non-endemic equilibrium points in the equation (21) will be stable if $R_0<1$.

#### Proof

The stability of the endemic equilibrium point is seen from the eigenvalues generated from the characteristic equation of the model in equations (1) to (9). The characteristic equation at the endemic equilibrium points (21) is obtained as in the equation (25).

25 $$\begin{aligned} \frac{1}{rK(\phi \mu _2 V)^2)^2}((\lambda +q_2+\rho )(\lambda +r+q_0)(B(\mu +\mu _1-\xi -\mu _2 V\phi )-\mu _2 V \phi (f+\lambda ))P_3(\lambda )P_4(\lambda )P_5(\lambda ))=0 \end{aligned}$$

with $$P_1(\lambda )=a_0\lambda ^2+a_1\lambda +a_2, P_2(\lambda )=a_3\lambda ^2+a_4\lambda +a_5, P_3(\lambda )=a_6\lambda ^2+a_7\lambda +a_8,$$ and

26 $$\begin{aligned} a_0&= -\mu _2 V \phi < 0 \end{aligned}$$

27 $$\begin{aligned} a_1&= B(\xi - (\mu + \mu _1)) < 0 \end{aligned}$$

28 $$\begin{aligned} a_2&= -(\mu +\mu _1-\xi )\left[ V \phi \mu _2 (B - (\mu +\mu _1)) - B(\mu +\mu _1 - \xi )\right] < 0 \end{aligned}$$

29 $$\begin{aligned} a_3&= -\mu _2 K V \phi r < 0 \end{aligned}$$

30 $$\begin{aligned} a_4&= -rK\left[ B(\xi - \mu - \mu _1) + \mu _2 V \phi (B + \rho + q_1)\right] < 0 \end{aligned}$$

31 $$\begin{aligned} a_5&= \beta b V (r + q_0)(\mu + \mu _1 - \xi ) + K B (q_1 + \rho )\left[ \mu + \mu _1 - \xi - \mu _2 V \phi \right]< 0 \nonumber \\&= R_{01}^2 - 1 < 0 \end{aligned}$$

32 $$\begin{aligned} a_6&= -\mu _2 K V \phi r < 0 \end{aligned}$$

33 $$\begin{aligned} a_7&= -rK\left[ \mu _2 V \phi (B + q_3 + c + \rho ) - B(\xi - \mu - \mu _1)\right] < 0 \end{aligned}$$

34 $$\begin{aligned} a_8&= a \alpha V (q_0 + r)(\mu + \mu _1 - \xi ) + K r (\rho + q_3)\left[ B(\mu + \mu _1 - \xi ) - \mu _2 \phi V (B + c)\right]< 0 \nonumber \\&= R_{02}^2 - 1 < 0 \end{aligned}$$

From the equation (26) to (34) it can be seen that the system will be stable at the endemic equilibrium point if $R_0 < 1$; $R_0 = \max \{ R_{01}, R_{02} \}$.$\square$

## Sensitivity analysis

**Fig. 3:**
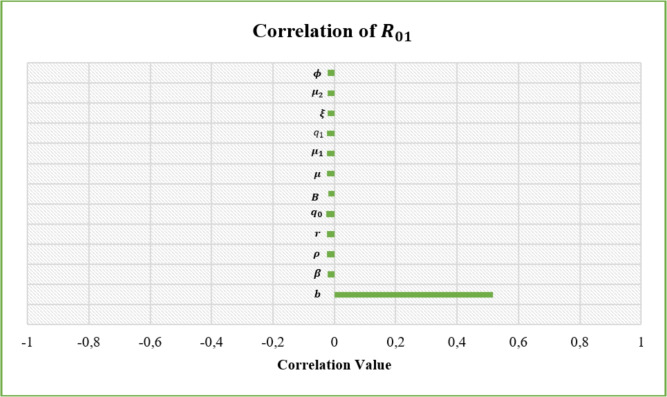
Correlation value for $R_{01}$.

**Fig. 4:**
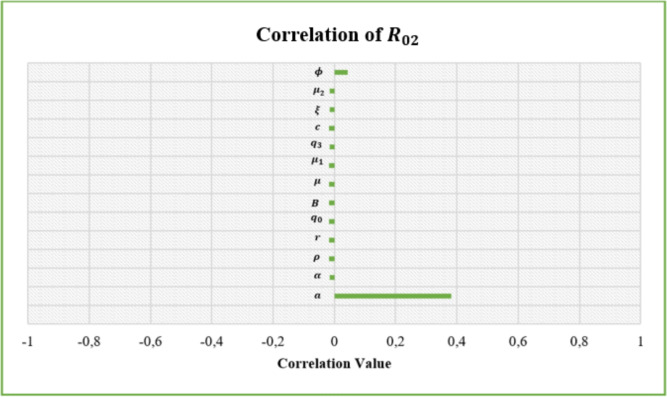
Correlation value for $R_{02}$.

Sensitivity analysis was conducted using the Latin Hypercube Sampling (LHS) method and the Partial Rank Correlation Coefficient (PRCC) method^36^. A total of 5000 samples were used to determine the parameters that affect the basic reproduction rate, where each parameter is assumed to have a value between 0 and 1. The results obtained from the sensitivity analysis are presented in Table 2, Figs. 3 and 4.

**Table 2:** Sensitivity Analysis.

Parameter	Correlation of $R_{01}$	Parameter	Correlation of $R_{02}$
*b*	0.516649122507620	*a*	0.382233097801835
$\beta$	$-0.209078683084698e^{-1}$	$\alpha$	$-0.153233489263352e^{-1}$
$\rho$	−0.237288202875208e-1	$\rho$	−0.162343551016891e-1
$q_0$	−0.256416546763217e-1	$q_0$	−0.175091013662951e-1
*B*	−0.190342087431791e-1	*B*	−0.163267789633680e-1
$\mu$	−0.244443981866709e-1	$\mu$	−0.169036501797706e-1
$\mu _1$	−0.252103994064210e-1	$\mu _1$	−0.172979654050245e-1
$q_1$	−0.232677643583619e-1	$q_3$	−0.156956559186890e-1
$\xi$	−0.222130150914830e-1	*c*	−0.167546078065443e-1
$\mu _2$	−0.214825595430636e-1	$\xi$	−0.161689938256355e-1
$\phi$	−0.227714901901263e-1	$\mu _2$	−0.161689938256355e-1
		$\phi$	0.0442179092085094

The results of the global sensitivity analysis (as written in Table 2) show that parameter a is positively correlated with $R_{02}$. In contrast, parameter b positively correlates with $R_{01}$, while the other parameters negatively correlate with $R_{0}$ (both with $R_{01}$ and $R_{02}$). This indicates that the value of $R_{0}$ will be more significant if the values of the parameters *a* and *b* are greater, and vice versa. In other words, the infectiousness of tungro virus disease is greatly influenced by the rate of transition of susceptible vectors to RTSV-infected vectors after taking food from RTSV-infected rice plants and the transition rate of susceptible vectors to RTSV+RTBV-infected vectors after taking food from RTSV+RTBV-infected rice plants.

## Optimal control

The optimal control strategy discussed in this section aims to minimize the objective function defined in the model. This objective function accounts for both the costs of implementing control measures–namely, roguing, insecticide application, and the conservation of natural enemies through the planting of refugia plants–and the losses in crop yield caused by rice tungro disease.

The optimal control is analyzed through seven different scenarios involving three types of control measures: (1) roguing, (2) insecticides, and (3) conservation of natural enemies. The first three scenarios apply each control method individually, while the following three scenarios explore pairwise combinations: roguing with natural enemies, roguing with insecticides, and natural enemies with insecticides. These scenarios are designed to identify the most effective strategy for reducing the spread of rice tungro disease.

The goal of the optimal control model is to minimize both the population of rice plants and vector insects infected with RTSV or co-infected with RTSV and RTBV, as well as to reduce the economic costs incurred by farmers. The corresponding optimal control problem is formulated as shown in Equation (35).

35 $$\begin{aligned} J(u_r, u_p, u_M) = \min \int _{t_0}^{t_1} \left( A_1 P_1 + A_2 P_3 + A_3 V_1 + A_4 V_3 + C_1 u_r^2 + C_2 u_p^2 + C_3 u_M^2 \right) \, dt \end{aligned}$$

### Theorem 5

For $t \in [0, t_1]$, there exists an optimal control $u^* = (u_r, u_p, u_M)$ that corresponds to a suitable state trajectory such that it controls the initial value of the constraint function while minimizing the objective functional over the admissible control set $\zeta$.

### Proof

The proof follows from the optimal control theory results in *Fleming & Rishel (2012)*, which state that an optimal control exists if the following conditions are satisfied:

(i) The set of admissible controls and corresponding state trajectories is non-empty.
(ii) The right-hand side of the control system is linear in the control variables.
(iii) The integrand *L*(*t*, *x*, *u*) is convex in *u*.

*Condition (i):*

The right-hand side of the controlled system is given by

$$\frac{dx}{dt} = K(t, x, u),$$

where *K*(*t*, *x*, *u*) is a bounded and continuous function. Therefore, a unique solution exists for the initial value problem by the Picard–Lindelöf theorem (Schroers, 2011), implying the admissible set is non-empty.

*Condition (ii):*

The dynamics can be written as:

$$G(t, x, u) = K(t, x, u) + B(x, t) \cdot u,$$

where *B*(*x*, *t*) is a matrix that multiplies the control vector $u = (u_r, u_p, u_M)^\top$. Since *G*(*t*, *x*, *u*) is linear in *u*, condition (ii) is satisfied.

*Condition (iii):*

We consider the objective integrand:

$$L(t,x,u) = \sum _{i=0}^{3} P_i(t) + \sum _{j=0}^{3} V_j(t) +M_A(t)+ C_1 u_r^2 + C_2 u_p^2 + C_3 u_M^2.$$

Let $u, v \in \zeta$ and $\xi \in (0,1)$. Then,

$$(1 - \xi ) L(t, x, u) + \xi L(t, x, v) = \sum _{i=0}^{3} P_i(t) + \sum _{j=0}^{3} V_j(t) +M_A(t)+ (1-\xi )(C_1 u_r^2 + C_2 u_p^2 + C_3 u_M^2) + \xi (C_1 v_r^2 + C_2 v_p^2 + C_3 v_M^2),$$

and

$$L(t, x, (1 - \xi )u + \xi v) = \sum _{i=0}^{3} P_i(t) + \sum _{j=0}^{3} V_j(t)+M_A(t) + C_1((1-\xi ) u_r + \xi v_r)^2 + C_2((1-\xi ) u_p + \xi v_p)^2 + C_3((1-\xi ) u_M + \xi v_M)^2.$$

The difference is

$$\Delta = (1 - \xi )L(t, x, u) + \xi L(t, x, v) - L(t, x, (1 - \xi )u + \xi v),$$

which simplifies to

$$\begin{aligned} \Delta&= C_1\Big [(1 - \xi ) u_r^2 + \xi v_r^2 - \big ((1 - \xi ) u_r + \xi v_r\big )^2\Big ] + C_2\Big [(1 - \xi ) u_p^2 + \xi v_p^2 - \big ((1 - \xi ) u_p + \xi v_p\big )^2\Big ] \\&\quad + C_3\Big [(1 - \xi ) u_M^2 + \xi v_M^2 - \big ((1 - \xi ) u_M + \xi v_M\big )^2\Big ]\\&= C_1 \, \xi (1 - \xi ) \begin{bmatrix} u_r&v_r \end{bmatrix} \begin{bmatrix} (1 - \xi )(1 - (1 - \xi )) & -\xi (1 - \xi ) \\ -\xi (1 - \xi ) & \xi (-\xi (1 - \xi )) \end{bmatrix} \begin{bmatrix} u_r \\ v_r \end{bmatrix} \\&\quad + C_2 \, \xi (1 - \xi ) \begin{bmatrix} u_p&v_p \end{bmatrix} \begin{bmatrix} (1 - \xi )(1 - (1 - \xi )) & -\xi (1 - \xi ) \\ -\xi (1 - \xi ) & \xi (-\xi (1 - \xi )) \end{bmatrix} \begin{bmatrix} u_p \\ v_p \end{bmatrix} \\&\quad + C_3 \, \xi (1 - \xi ) \begin{bmatrix} u_M&v_M \end{bmatrix} \begin{bmatrix} (1 - \xi )(1 - (1 - \xi )) & -\xi (1 - \xi ) \\ -\xi (1 - \xi ) & \xi (-\xi (1 - \xi )) \end{bmatrix} \begin{bmatrix} u_M \\ v_M \end{bmatrix}\\&= C_1 \, \xi (1 - \xi ) \begin{bmatrix} u_r&v_r \end{bmatrix} \begin{bmatrix} 1 & -1 \\ -1 & 1 \end{bmatrix} \begin{bmatrix} u_r \\ v_r \end{bmatrix} + C_2 \, \xi (1 - \xi ) \begin{bmatrix} u_p&v_p \end{bmatrix} \begin{bmatrix} 1 & -1 \\ -1 & 1 \end{bmatrix} \begin{bmatrix} u_p \\ v_p \end{bmatrix} \\&\quad + C_3 \, \xi (1 - \xi ) \begin{bmatrix} u_M&v_M \end{bmatrix} \begin{bmatrix} 1 & -1 \\ -1 & 1 \end{bmatrix} \begin{bmatrix} u_M \\ v_M \end{bmatrix} \ge 0 \end{aligned}$$

since quadratic functions are convex. Hence, *L*(*t*, *x*, *u*) is convex in *u*, and condition (iii) is satisfied. Therefore, the existence of an optimal control $u^* \in \zeta$ is guaranteed, and the theorem is proven.$\square$

The method used to solve the optimal control problem is the Pontryagin minimum principle, with $u_r$ being the control for roguing control, $u_p$ being the control for insecticide control, and $u_M$ being the control to conserve natural enemies (by planting refugia plants). The quadratic function in the objective function is used to measure the control cost, which assumes that there is no linear relationship between the impact of the intervention and the price of the intervention on the infected population (its inverse forms a nonlinear function). With constraint functions as in the equation (36) to (44)

36 $$\begin{aligned} \frac{dP_0}{dt}&= r(K-N_P)-\frac{\alpha P_{0}V_{3}}{N_{P}}-\frac{\gamma P_{0}V_{3}}{N_{P}}-\frac{\tau P_{0}V_{3}}{N_{P}}-\frac{\beta P_{0}V_{1}}{N_{P}}-\frac{\sigma P_{0}V_{2}}{N_{P}}-q_{0}P_{0}\end{aligned}$$

37 $$\begin{aligned} \frac{dP_{1}}{dt}&= \frac{\beta P_{0} V_{1}}{N_{P}}+\frac{\gamma P_{0} V_{1}}{N_{P}}-\frac{\lambda P_{1} V_{3}}{N_{P}}-q_{1} P_{1}-u_{r} P_{1}\end{aligned}$$

38 $$\begin{aligned} \frac{dP_{2}}{dt}&= \frac{\tau P_{0} V_{3}}{N_{P}}+\frac{\sigma P_{0} V_{2}}{N_{P}}-\frac{\delta P_{2} V_{3}}{N_{P}}-q_{2} P_{2}-u_{r} P_{2}\end{aligned}$$

39 $$\begin{aligned} \frac{dP_{3}}{dt}&= \frac{\alpha P_{0} V_{3}}{N_{P}}+\frac{\lambda P_{0} V_{3}}{N_{P}}-\frac{\delta P_{2} V_{3}}{N_{P}}-q_{3} P_{3}-u_{r} P_{3}\end{aligned}$$

40 $$\begin{aligned} \frac{dV_{0}}{dt}&= BN_{V}(1-\frac{N_{V}}{V})-\frac{a V_{0} P_{3}}{N_{P}}+\frac{b V_{0} P_{1}}{N_{P}}+f V_{2}-(\mu _{2} M_{A}+u_{p}+\mu ) V_{0}\end{aligned}$$

41 $$\begin{aligned} \frac{dV_1}{dt}&= \frac{bV_{0}P_{1}}{N_P}-\frac{gV_{1}P_{2}}{N_P}-(\mu _{2} M_{A}+u_{p}+\mu ) V_{1}\end{aligned}$$

42 $$\begin{aligned} \frac{dV_2}{dt}&=-fV_2+cV_3-(\mu _{2} M_{A}+u_{p}+\mu ) V_{2}\end{aligned}$$

43 $$\begin{aligned} \frac{dV_3}{dt}&= \frac{V_{0}P_{3}}{N_P}-\frac{gV_{1}P_{2}}{N_P}-cV_3-(\mu _{2} M_{A}+u_{p}+\mu ) V_{3}\end{aligned}$$

44 $$\begin{aligned} \frac{dM_A}{dt}&= u_{M} \phi \mu _{2} M_{A} N_{V}+\xi M_{A}-(\mu +u_{p})M_A \end{aligned}$$

Boundary condition: with $P_i(0),V_i(0),M_A(0)\ge 0;i=0, 1, 2, 3.$

From the objective function and constraints in equations (35) to (44), the Hamiltonian function is obtained as in equation (45).

45 $$\begin{aligned}&H=A_1P_1+A_2P_3+A_3V_1+A_4V_3+C_1u_r^2+C_2u_p^2+C_3u_M^2+\lambda _1\frac{dP_0}{dt}\nonumber \\&+\lambda _2\frac{dP_1}{dt}+\lambda _3\frac{dP_2}{dt}+\lambda _4\frac{dP_3}{dt}+\lambda _5\frac{dV_0}{dt}+\lambda _6\frac{dV_1}{dt}\nonumber \\&+\lambda _7\frac{dV_2}{dt}+\lambda _8\frac{dV_3}{dt}+\lambda _9\frac{dM_A}{dt} \end{aligned}$$

With $\lambda _i$, $i = 1, \dots , 9$, denoting the co-state variables, the Hamiltonian function must therefore satisfy the co-state, adjoint, and stationary conditions.

Co-state:

$$\begin{aligned} \frac{dP_0}{dt}&= r(K-N_P)-\frac{\alpha P_{0}V_{3}}{N_{P}}-\frac{\gamma P_{0}V_{3}}{N_{P}}-\frac{\tau P_{0}V_{3}}{N_{P}}-\frac{\beta P_{0}V_{1}}{N_{P}}-\frac{\sigma P_{0}V_{2}}{N_{P}}-q_{0}P_{0}\\ \frac{dP_{1}}{dt}&= \frac{\beta P_{0} V_{1}}{N_{P}}+\frac{\gamma P_{0} V_{1}}{N_{P}}-\frac{\lambda P_{1} V_{3}}{N_{P}}-q_{1} P_{1}-u_{r} P_{1}\\ \frac{dP_{2}}{dt}&= \frac{\tau P_{0} V_{3}}{N_{P}}+\frac{\sigma P_{0} V_{2}}{N_{P}}-\frac{\delta P_{2} V_{3}}{N_{P}}-q_{2} P_{2}-u_{r} P_{2}\\ \frac{dP_{3}}{dt}&= \frac{\alpha P_{0} V_{3}}{N_{P}}+\frac{\lambda P_{0} V_{3}}{N_{P}}-\frac{\delta P_{2} V_{3}}{N_{P}}-q_{3} P_{3}-u_{r} P_{3}\\ \frac{dV_{0}}{dt}&= BN_{V}(1-\frac{N_{V}}{V})-\frac{a V_{0} P_{3}}{N_{P}}+\frac{b V_{0} P_{1}}{N_{P}}+f V_{2}-(\mu _{2} M_{A}+u_{p}+\mu ) V_{0}\\ \frac{dV_1}{dt}&= \frac{bV_{0}P_{1}}{N_P}-\frac{gV_{1}P_{2}}{N_P}-(\mu _{2} M_{A}+u_{p}+\mu ) V_{1}\\ \frac{dV_2}{dt}&=-fV_2+cV_3-(\mu _{2} M_{A}+u_{p}+\mu ) V_{2}\\ \frac{dV_3}{dt}&= \frac{V_{0}P_{3}}{N_P}-\frac{gV_{1}P_{2}}{N_P}-cV_3-(\mu _{2} M_{A}+u_{p}+\mu ) V_{3}\\ \frac{dM_A}{dt}&= u_{M} \phi \mu _{2} M_{A} N_{V}+\xi M_{A}-(\mu +u_{p})M_{A} \end{aligned}$$

with $$P_i(0),V_i(0),M_A(0)\ge 0;i=0, 1, 2, 3.$$

Adjoint:

$$\begin{aligned} \dot{\lambda _1} =&- \lambda _1 \Bigg ( -r - \frac{\alpha V_3}{P_0 + P_1 + P_2 + P_3} + \frac{\alpha P_0 V_3}{(P_0 + P_1 + P_2 + P_3)^2}- \frac{\gamma V_3}{P_0 + P_1 + P_2 + P_3}+ \frac{\gamma P_0 V_3}{(P_0 + P_1 + P_2 + P_3)^2}\\&- \frac{\tau V_3}{P_0 + P_1 + P_2 + P_3} + \frac{\tau P_0 V_3}{(P_0 + P_1 + P_2 + P_3)^2} - \frac{\beta V_1}{P_0 + P_1 + P_2 + P_3} + \frac{\beta P_0 V_1}{(P_0 + P_1 + P_2 + P_3)^2}- \frac{\sigma V_2}{P_0 + P_1 + P_2 + P_3}\\&+ \frac{\sigma P_0 V_2}{(P_0 + P_1 + P_2 + P_3)^2} - q_0 \Bigg ) - \lambda _2 \Bigg ( \frac{\beta V_1}{P_0 + P_1 + P_2 + P_3} - \frac{\beta P_0 V_1}{(P_0 + P_1 + P_2 + P_3)^2} + \frac{\gamma V_3}{P_0 + P_1 + P_2 + P_3}\\&- \frac{\gamma P_0 V_3}{(P_0 + P_1 + P_2 + P_3)^2} + \frac{\lambda P_1 V_3}{(P_0 + P_1 + P_2 + P_3)^2} \Bigg ) - \lambda _3 \Bigg ( \frac{\tau V_3}{P_0 + P_1 + P_2 + P_3} - \frac{\tau P_0 V_3}{(P_0 + P_1 + P_2 + P_3)^2}\\&+ \frac{\sigma V_2}{P_0 + P_1 + P_2 + P_3}- \frac{\sigma P_0 V_2}{(P_0 + P_1 + P_2 + P_3)^2} + \frac{\delta P_2 V_3}{(P_0 + P_1 + P_2 + P_3)^2} \Bigg ) - \lambda _4 \Bigg ( \frac{\alpha V_3}{P_0 + P_1 + P_2 + P_3}\\&- \frac{\alpha P_0 V_3}{(P_0 + P_1 + P_2 + P_3)^2}- \frac{\lambda P_1 V_3}{(P_0 + P_1 + P_2 + P_3)^2}- \frac{\delta P_2 V_3}{(P_0 + P_1 + P_2 + P_3)^2} \Bigg ) - \lambda _5 \Bigg ( \frac{a V_0 P_3}{(P_0 + P_1 + P_2 + P_3)^2}+\\&\frac{b V_0 P_1}{(P_0 + P_1 + P_2 + P_3)^2} \Bigg )-\lambda _6 \Bigg ( - \frac{b V_0 P_1}{(P_0 + P_1 + P_2 + P_3)^2} + \frac{g V_1 P_2}{(P_0 + P_1 + P_2 + P_3)^2} \Bigg )- \lambda _8 \Bigg ( - \frac{a V_0 P_3}{(P_0 + P_1 + P_2 + P_3)^2}\\&-\frac{g V_1 P_2}{(P_0 + P_1 + P_2 + P_3)^2} \Bigg )\\ \end{aligned}$$

$$\begin{aligned} \dot{\lambda _2} =&-A_1 - \lambda _1 \Bigg ( -r + \frac{\alpha P_0 V_3}{(P_0 + P_1 + P_2 + P_3)^2} + \frac{\gamma P_0 V_3}{(P_0 + P_1 + P_2 + P_3)^2} + \frac{\tau P_0 V_3}{(P_0 + P_1 + P_2 + P_3)^2} + \frac{\beta P_0 V_1}{(P_0 + P_1 + P_2 + P_3)^2}\\&+ \frac{\sigma P_0 V_2}{(P_0 + P_1 + P_2 + P_3)^2} \Bigg ) - \lambda _2 \Bigg ( - \frac{\beta P_0 V_1}{(P_0 + P_1 + P_2 + P_3)^2} - \frac{\gamma P_0 V_3}{(P_0 + P_1 + P_2 + P_3)^2} - \frac{\lambda V_3}{P_0 + P_1 + P_2 + P_3} - q_1 - u_2\\&+ \frac{\lambda P_1 V_3}{(P_0 + P_1 + P_2 + P_3)^2} \Bigg ) - \lambda _3 \Bigg ( - \frac{\tau P_0 V_3}{(P_0 + P_1 + P_2 + P_3)^2} - \frac{\sigma P_0 V_2}{(P_0 + P_1 + P_2 + P_3)^2} + \frac{\delta P_2 V_3}{(P_0 + P_1 + P_2 + P_3)^2} \Bigg ) \\&- \lambda _4 \Bigg ( - \frac{\alpha P_0 V_3}{(P_0 + P_1 + P_2 + P_3)^2} + \frac{\lambda V_3}{P_0 + P_1 + P_2 + P_3} - \frac{\lambda P_1 V_3}{(P_0 + P_1 + P_2 + P_3)^2} - \frac{\delta P_2 V_3}{(P_0 + P_1 + P_2 + P_3)^2} \Bigg )\\&- \lambda _5 \Bigg ( \frac{a V_0 P_3}{(P_0 + P_1 + P_2 + P_3)^2} - \frac{b V_0}{P_0 + P_1 + P_2 + P_3} + \frac{b V_0 P_1}{(P_0 + P_1 + P_2 + P_3)^2} \Bigg ) - \lambda _6 \Bigg ( \frac{b V_0}{P_0 + P_1 + P_2 + P_3}\\&- \frac{b V_0 P_1}{(P_0 + P_1 + P_2 + P_3)^2} + \frac{g V_1 P_2}{(P_0 + P_1 + P_2 + P_3)^2} \Bigg ) - \lambda _8 \Bigg ( - \frac{a V_0 P_3}{(P_0 + P_1 + P_2 + P_3)^2} - \frac{g V_1 P_2}{(P_0 + P_1 + P_2 + P_3)^2} \Bigg )\\ \end{aligned}$$

$$\begin{aligned} \dot{\lambda _3} =&-{ \lambda _1} \left( -r + \frac{\alpha P_{0} V_{3}}{(P_{0} + P_{1} + P_{2} + P_{3})^2} + \frac{\gamma P_{0} V_{3}}{(P_{0} + P_{1} + P_{2} + P_{3})^2} + \frac{\tau P_{0} V_{3}}{(P_{0} + P_{1} + P_{2} + P_{3})^2} \right. + \frac{\beta P_{0} V_{1}}{(P_{0} + P_{1} + P_{2} + P_{3})^2} \\&+ \frac{\sigma P_{0} V_{2}}{(P_{0} + P_{1} + P_{2} + P_{3})^2} \Big )- { \lambda _2} \left( - \frac{\beta P_{0} V_{1}}{(P_{0} + P_{1} + P_{2} + P_{3})^2} - \frac{\gamma P_{0} V_{3}}{(P_{0} + P_{1} + P_{2} + P_{3})^2} + \frac{\lambda P_{1} V_{3}}{(P_{0} + P_{1} + P_{2} + P_{3})^2} \right) \\&- { \lambda _3} \left( - \frac{\tau P_{0} V_{3}}{(P_{0} + P_{1} + P_{2} + P_{3})^2} - \frac{\sigma P_{0} V_{2}}{(P_{0} + P_{1} + P_{2} + P_{3})^2} \right. \left. - \frac{\delta V_{3}}{P_{0} + P_{1} + P_{2} + P_{3}} + \frac{\delta P_{2} V_{3}}{(P_{0} + P_{1} + P_{2} + P_{3})^2} - q_{2} - u_{2} \right) \\&- { \lambda _4} \left( - \frac{\alpha P_{0} V_{3}}{(P_{0} + P_{1} + P_{2} + P_{3})^2} - \frac{\lambda P_{1} V_{3}}{(P_{0} + P_{1} + P_{2} + P_{3})^2} \right. \left. + \frac{\delta V_{3}}{P_{0} + P_{1} + P_{2} + P_{3}} - \frac{\delta P_{2} V_{3}}{(P_{0} + P_{1} + P_{2} + P_{3})^2} \right) \\&- { \lambda _5} \left( \frac{a V_{0} P_{3}}{(P_{0} + P_{1} + P_{2} + P_{3})^2} + \frac{b V_{0} P_{1}}{(P_{0} + P_{1} + P_{2} + P_{3})^2} \right) - { \lambda _6} \left( - \frac{b V_{0} P_{1}}{(P_{0} + P_{1} + P_{2} + P_{3})^2} - \frac{g V_{1}}{P_{0} + P_{1} + P_{2} + P_{3}} \right. \\&\left. + \frac{g V_{1} P_{2}}{(P_{0} + P_{1} + P_{2} + P_{3})^2} \right) - { \lambda _8} \left( - \frac{a V_{0} P_{3}}{(P_{0} + P_{1} + P_{2} + P_{3})^2} + \frac{g V_{1}}{P_{0} + P_{1} + P_{2} + P_{3}} \right. \left. - \frac{g V_{1} P_{2}}{(P_{0} + P_{1} + P_{2} + P_{3})^2} \right) \end{aligned}$$

$$\begin{aligned} \dot{\lambda _4} ={}&-A_2 - \lambda _1 \bigg ( -r + \frac{\alpha P_0 V_3}{(P_0 + P_1 + P_2 + P_3)^2} + \frac{\gamma P_0 V_3}{(P_0 + P_1 + P_2 + P_3)^2} + \frac{\tau P_0 V_3}{(P_0 + P_1 + P_2 + P_3)^2} + \frac{\beta P_0 V_1}{(P_0 + P_1 + P_2 + P_3)^2}\\&+ \frac{\sigma P_0 V_2}{(P_0 + P_1 + P_2 + P_3)^2} \bigg ) - \lambda _2 \bigg ( - \frac{\beta P_0 V_1}{(P_0 + P_1 + P_2 + P_3)^2} - \frac{\gamma P_0 V_3}{(P_0 + P_1 + P_2 + P_3)^2} + \frac{\lambda P_1 V_3}{(P_0 + P_1 + P_2 + P_3)^2} \bigg )\\&- \lambda _3 \bigg ( - \frac{\tau P_0 V_3}{(P_0 + P_1 + P_2 + P_3)^2} - \frac{\sigma P_0 V_2}{(P_0 + P_1 + P_2 + P_3)^2} + \frac{\delta P_2 V_3}{(P_0 + P_1 + P_2 + P_3)^2} \bigg ) - \lambda _4 \bigg ( - \frac{\alpha P_0 V_3}{(P_0 + P_1 + P_2 + P_3)^2}\\&- \frac{\lambda P_1 V_3}{(P_0 + P_1 + P_2 + P_3)^2} - \frac{\delta P_2 V_3}{(P_0 + P_1 + P_2 + P_3)^2} - q_3 - u_2 \bigg ) - \lambda _5 \bigg ( - \frac{aV_0}{P_0 + P_1 + P_2 + P_3} + \frac{aV_0 P_3}{(P_0 + P_1 + P_2 + P_3)^2}\\&+ \frac{bV_0 P_1}{(P_0 + P_1 + P_2 + P_3)^2} \bigg ) - \lambda _6 \bigg ( - \frac{bV_0 P_1}{(P_0 + P_1 + P_2 + P_3)^2} + \frac{gV_1 P_2}{(P_0 + P_1 + P_2 + P_3)^2} \bigg ) - \lambda _8 \bigg ( \frac{aV_0}{P_0 + P_1 + P_2 + P_3}\\&- \frac{aV_0 P_3}{(P_0 + P_1 + P_2 + P_3)^2} - \frac{gV_1 P_2}{(P_0 + P_1 + P_2 + P_3)^2} \bigg ) \end{aligned}$$

$$\begin{aligned} \dot{\lambda _5} ={}&-\lambda _5 \bigg ( B \left( 1 - \frac{V_0 + V_1 + V_2 + V_3}{V} \right) - \frac{B (V_0 + V_1 + V_2 + V_3)}{V} - \frac{aP_3}{P_0 + P_1 + P_2 + P_3} - \frac{bP_1}{P_0 + P_1 + P_2 + P_3} - \mu - u_3 \\&- \mu _2 M_A\bigg )- \frac{\lambda _6\, bP_1}{P_0 + P_1 + P_2 + P_3} - \frac{\lambda _8\, aP_3}{P_0 + P_1 + P_2 + P_3} - \lambda _9\, \phi \, \mu _2\, M_A \end{aligned}$$

$$\begin{aligned} \dot{\lambda _6} ={}&\frac{\lambda _1\, \beta \, P_0}{P_0 + P_1 + P_2 + P_3} - \frac{\lambda _2\, \beta \, P_0}{P_0 + P_1 + P_2 + P_3} - \lambda _5 \bigg ( B \left( 1 - \frac{V_0 + V_1 + V_2 + V_3}{V} \right) - \frac{B (V_0 + V_1 + V_2 + V_3)}{V} \bigg ) - \lambda _6 \bigg (- \mu \\&- \frac{gP_2}{P_0 + P_1 + P_2 + P_3} - \mu _2 M_A - u_3 \bigg ) - \frac{\lambda _8\, gP_2}{P_0 + P_1 + P_2 + P_3} - \lambda _9\, \phi \, \mu _2\, M_A \end{aligned}$$

$$\begin{aligned} \dot{\lambda _7} ={}&- A_3 + \frac{\lambda _1\, \sigma \, P_0}{P_0 + P_1 + P_2 + P_3} - \frac{\lambda _3\, \sigma \, P_0}{P_0 + P_1 + P_2 + P_3} - \lambda _5 \bigg ( B \left( 1 - \frac{V_0 + V_1 + V_2 + V_3}{V} \right) - \frac{B (V_0 + V_1 + V_2 + V_3)}{V} + f \bigg ) \\&- \lambda _7 \big ( - \mu _2 M_A - f - u_3 - \mu \big ) - \lambda _9\, \phi \, \mu _2\, M_A \end{aligned}$$

$$\begin{aligned} \dot{\lambda _8} ={}&- A_4 - \lambda _1 \bigg ( - \frac{\alpha P_0}{P_0 + P_1 + P_2 + P_3} - \frac{\gamma P_0}{P_0 + P_1 + P_2 + P_3} - \frac{\tau P_0}{P_0 + P_1 + P_2 + P_3} \bigg ) - \lambda _2 \bigg ( \frac{\gamma P_0}{P_0 + P_1 + P_2 + P_3} \\&- \frac{\lambda P_1}{P_0 + P_1 + P_2 + P_3} \bigg ) - \lambda _3 \bigg ( \frac{\tau P_0}{P_0 + P_1 + P_2 + P_3} - \frac{\delta P_2}{P_0 + P_1 + P_2 + P_3} \bigg ) - \lambda _4 \bigg ( \frac{\alpha P_0}{P_0 + P_1 + P_2 + P_3} + \frac{\lambda P_1}{P_0 + P_1 + P_2 + P_3}\\&+ \frac{\delta P_2}{P_0 + P_1 + P_2 + P_3} \bigg ) - \lambda _5 \bigg ( B \left( 1 - \frac{V_0 + V_1 + V_2 + V_3}{V} \right) - \frac{B (V_0 + V_1 + V_2 + V_3)}{V} \bigg ) - \lambda _7\, c - \lambda _8 \big ( - \mu _2 M_A - c - u_3 \\&- \mu \big ) - \lambda _9\, \phi \, \mu _2\, M_A \end{aligned}$$

$$\begin{aligned} \dot{\lambda _9} ={}&\lambda _5\, \mu _2\, V_0 + \lambda _6\, \mu _2\, V_1 + \lambda _7\, \mu _2\, V_2 + \lambda _8\, \mu _2\, V_3 - \lambda _9 \big ( u_1 + \phi \, \mu _2\, (V_0 + V_1 + V_2 + V_3) - \mu - u_3 \big ) \end{aligned}$$

Stationary conditions: because $0 \le u_M,u_p, u_r \le 1$, so:

$$\begin{aligned} u_r^* = \max \left\{ 0, \min \left( 1, \frac{1}{2} \cdot \frac{ \lambda _2(t) P_1(t) + \lambda _3(t) P_2(t) + \lambda _4(t) P_3(t) }{C_1} \right) \right\} \end{aligned}$$

$$\begin{aligned} u_P^* = \max \left\{ 0, \min \left( 1, \frac{1}{2} \cdot \frac{ \lambda _5(t) V_0(t) + \lambda _6(t) V_1(t) + \lambda _7(t) V_2(t) + \lambda _8(t) V_3(t) + \lambda _9(t) M_A(t) }{C_2} \right) \right\} \end{aligned}$$

$$\begin{aligned} u_M^* = \max \left\{ 0, \min \left( 1, -\frac{1}{2} \frac{\lambda _9(t) \, M_A(t)}{C_3} \right) \right\} \end{aligned}.$$

## Numerical simulation

### Numerical simulation of model

**Fig. 5:**
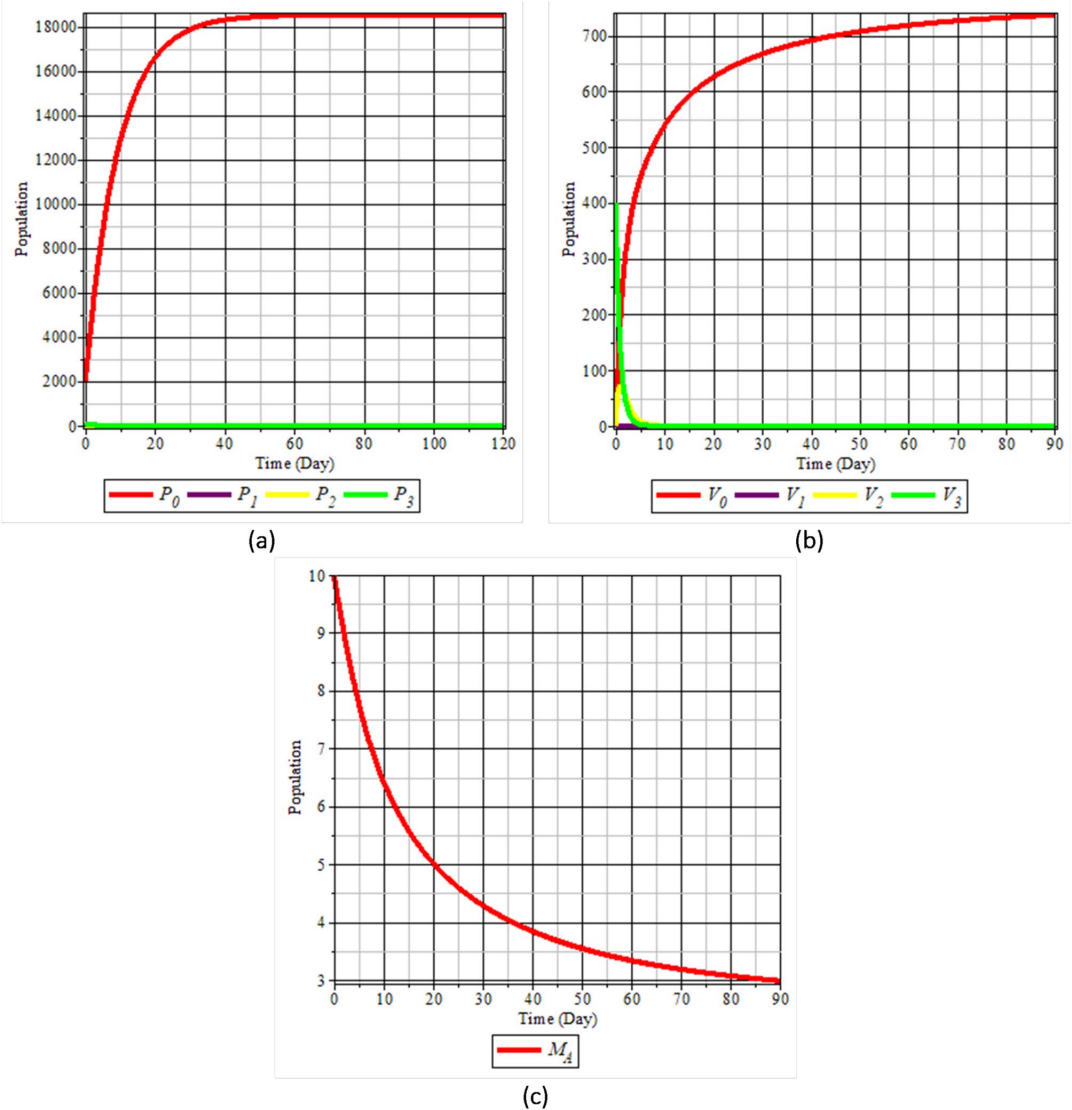
Population dynamics when $R_0<1$: (**a**) rice plants; (**b**) vectors; and (**c**) natural enemies.

**Fig. 6:**
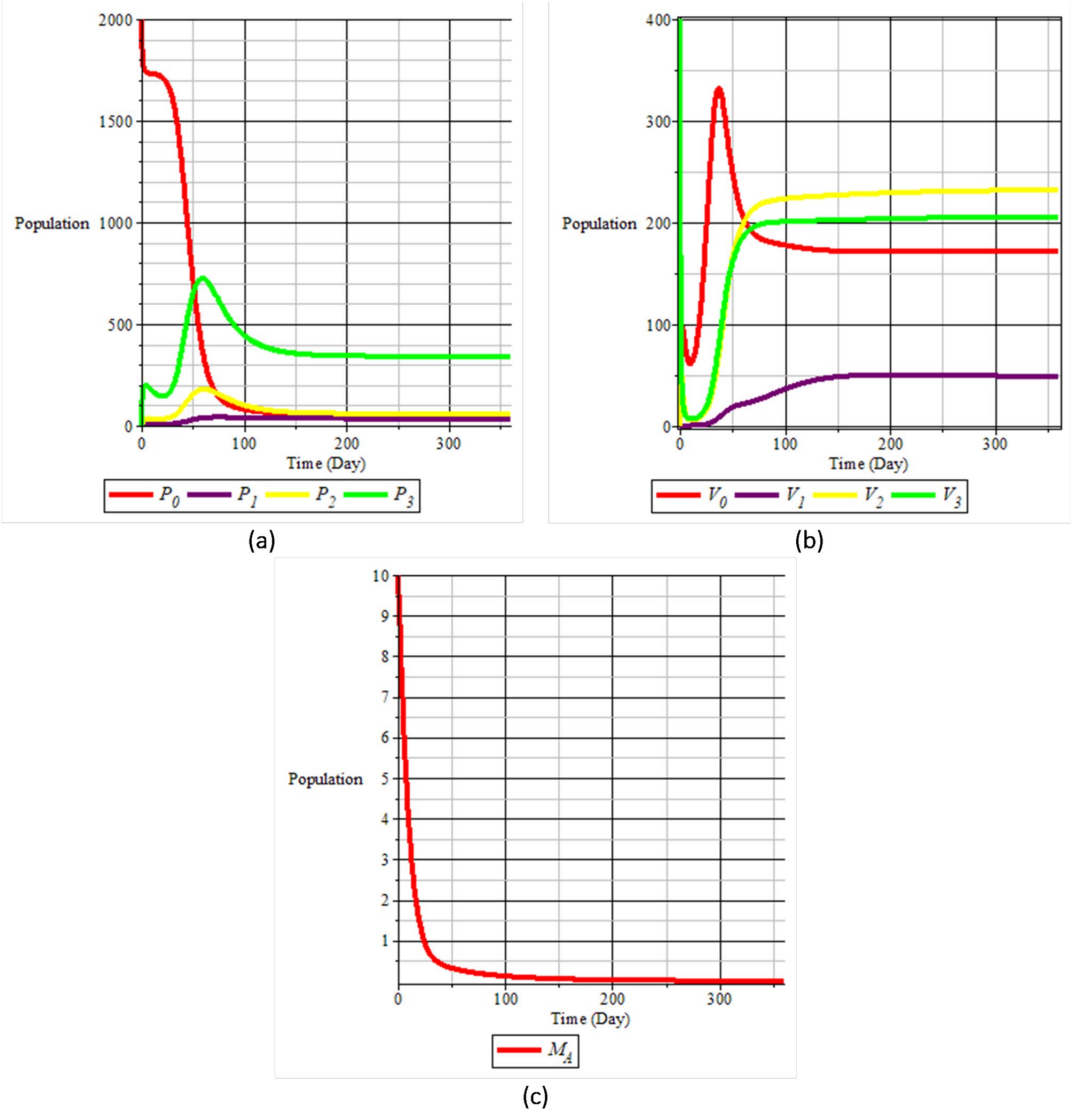
Population dynamics when $R_0>1$: (**a**) rice plants; (**b**) vectors; and (**c**) natural enemies.

This numerical simulation is performed to support the results of the dynamic analysis in the previous point. Figs. 5 to 6 are the results of numerical simulations to study the population dynamics of the mathematical model of the spread of rice tungro disease. The parameter values used in the numerical simulation to study the dynamics of this population are based on the initial values, and the parameters used are shown in Table 1

Figure 5 shows the simulation results when $R_0 < 1$. The healthy plant population $P_0$ increases steadily over time, while the infected subpopulations $P_1$, $P_2$ and $P_3$ decrease to zero. Similarly, the uninfected vector population $V_0$ dominates, whereas all subpopulations of infected vectors ($V_1$, $V_2$, $V_3$) vanish as time progresses. The alternate host population $M_A$ also decreases continuously. These results indicate that the disease eventually dies out and the system approaches a disease-free equilibrium. This suggests that the disease-free equilibrium is stable when $R_0 < 1$, in line with the theoretical expectation.

Figure 6, on the other hand, presents the dynamics when $R_0> 1$. In this case, the infected plant populations ($P_1$, $P_2$, $P_3$) and infected vector populations ($V_1$, $V_2$, $V_3$) persist over time at positive levels, indicating the existence of an endemic equilibrium. Although the initial dynamics involve oscillations or transients, the system stabilizes to a steady state where the infection remains present in both plants and vectors. The alternate host population $M_A$ decreases again, but does not influence the eradication of the disease.

### Numerical simulation of optimal control

#### Case 1

**Fig. 7:**
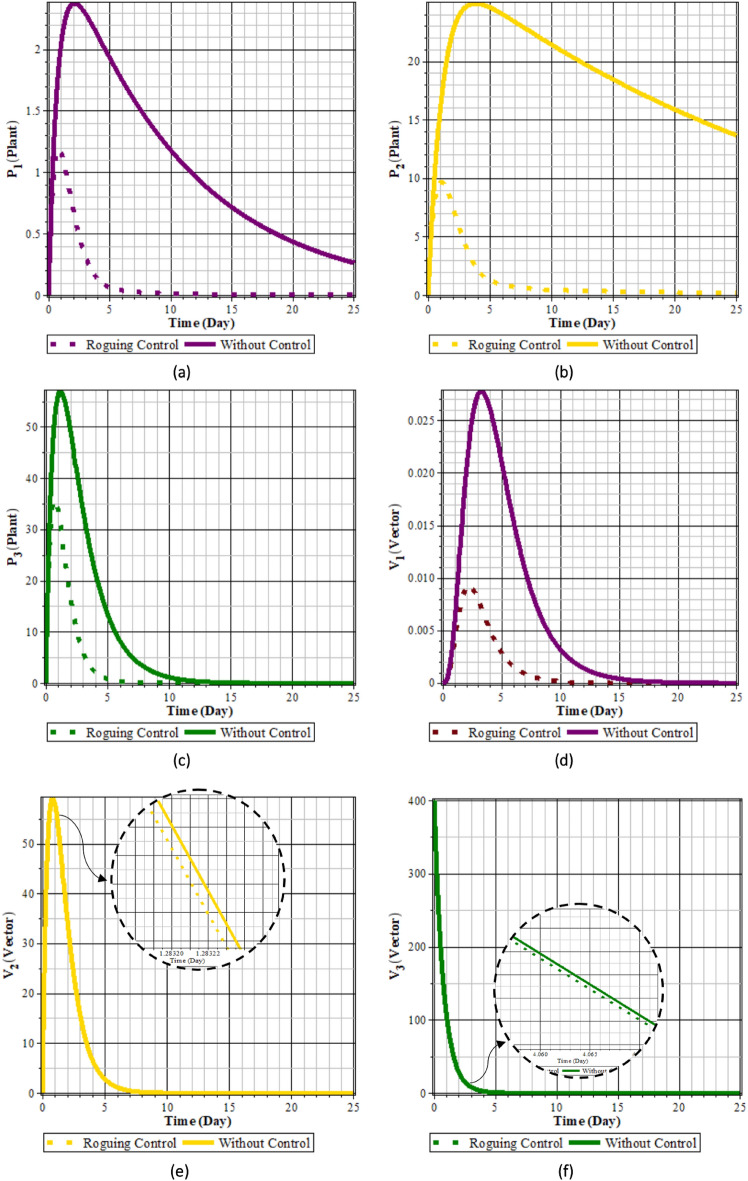
Comparison of populations using roguing control and without control: (**a**) rice plants infected with RTSV; (**b**) rice plants infected with RTBV; (**c**) rice plants infected with RTSV+RTBV; (**d**) green leafhopper vector infected with RTSV; (**e**) green leafhopper vector infected with RTBV; and (**f**) green leafhopper vector infected with RTSV+RTBV.

**Fig. 8:**
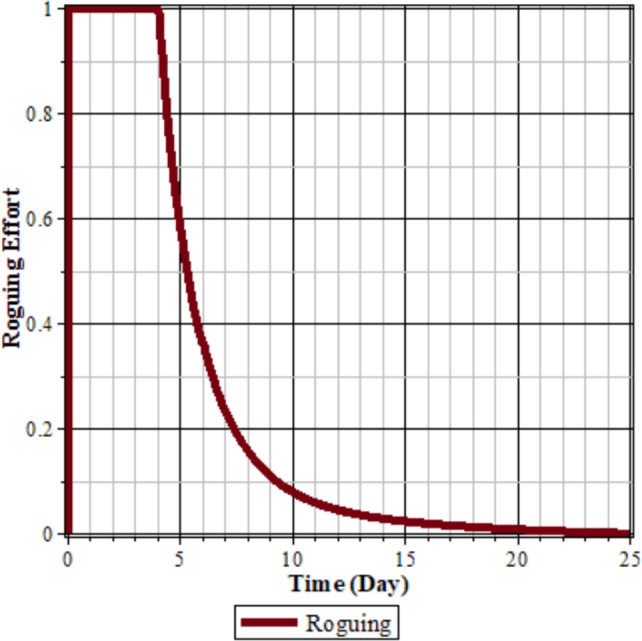
Roguing control.

From Figs. 7 to 8, it can be seen that in the simulation scenario, implementing 100% roguing of rice plants infected with RTSV, RTBV, and RTSV + RTBV every 5 days results in a reduction in the intensity of tungro disease spread. This is reflected in the decreased number of infected plants after roguing compared to before the control was applied. This intervention has a significant impact on the population of infected plants, although it has a minimal direct effect on the population of disease vectors (RTBV and RTSV + RTBV).

However, it is essential to note that the 100% roguing scenario in this model represents an idealized condition that does not accurately reflect real-world field situations. In practice, achieving complete removal of infected plants is highly unlikely due to various factors, such as delayed symptom detection, asymptomatic infections, and limited labor capacity. Previous studies have shown that the effectiveness of roguing in the field typically ranges from 70% to 90%, depending on detection accuracy and monitoring frequency^37^. Therefore, the results presented in this scenario should represent the maximum potential effectiveness of roguing.

#### Case 2

**Fig. 9:**
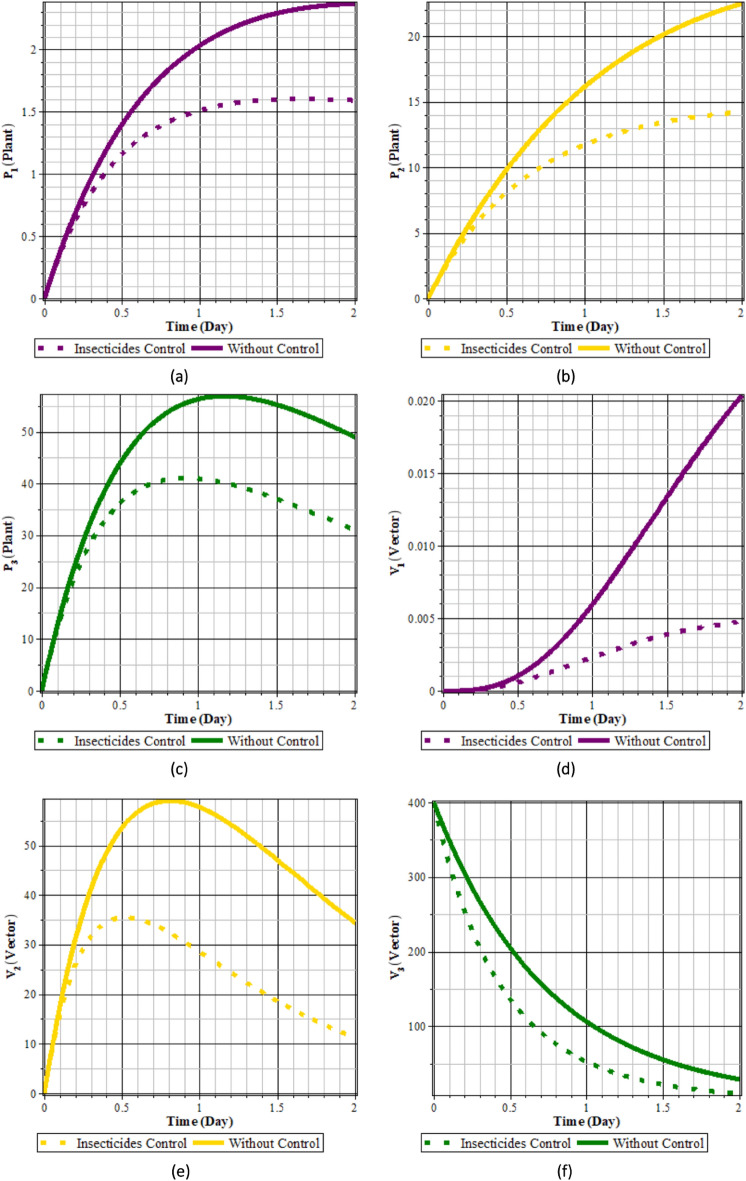
Comparison of populations using insecticide control and without control: (**a**) rice plants infected with RTSV; (**b**) rice plants infected with RTBV; (**c**) rice plants infected with RTSV+RTBV; (**d**) green leafhopper vector infected with RTSV; (**e**) green leafhopper vector infected with RTBV; and (**f**) green leafhopper vector infected with RTSV+RTBV.

**Fig. 10:**
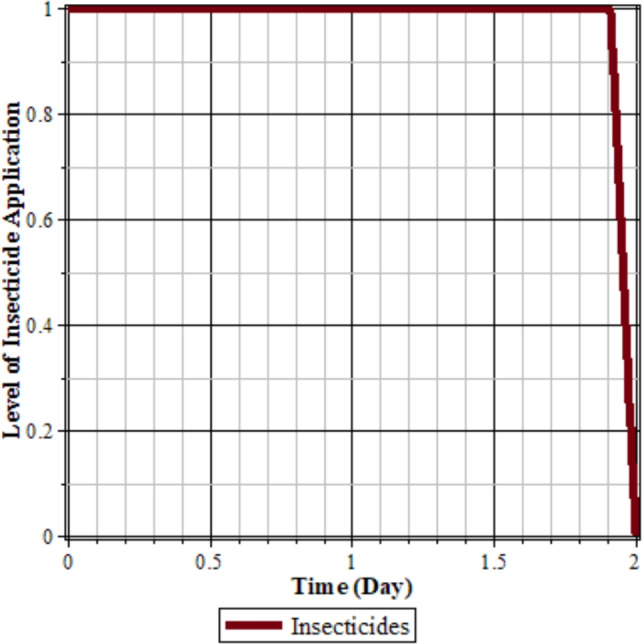
insecticide control.

From Figs. 9 to 10, it can be seen that the control by applying insecticide 100% for 2 days can reduce the intensity of the spread of rice tungro disease. This can be seen from the population of rice plants and vectors infected with RTSV, RTBV, and RTSV + RTBV before control in the form of insecticide application, which is more significant when compared to after control in the form of insecticide application. This control dramatically affects the population of infected plants and vectors. This happens because applying insecticides to plants causes death in vectors when the vectors take food from plants that have been applied with insecticides, so the vector population decreases.

#### Case 3

**Fig. 11:**
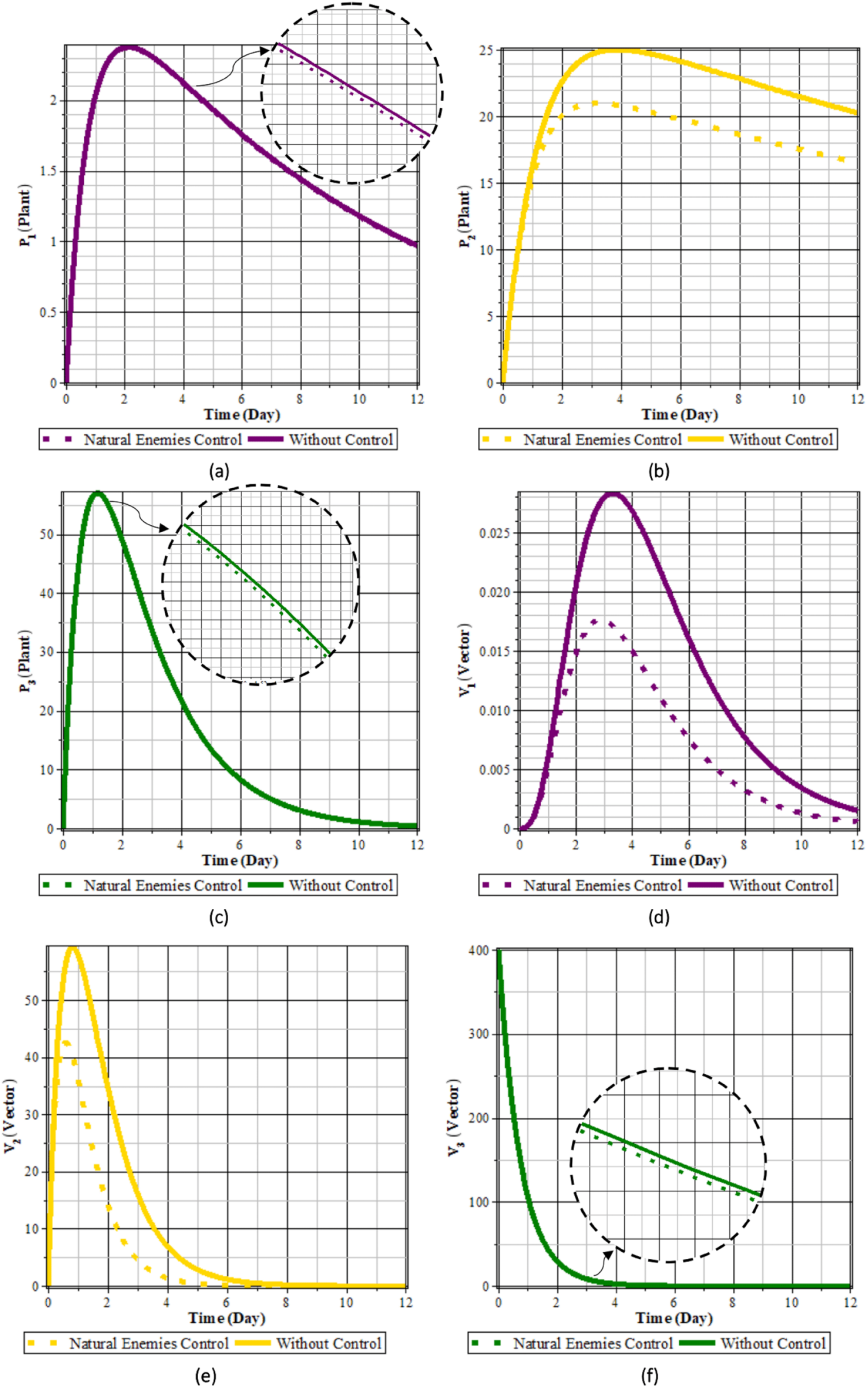
Comparison of populations using natural enemies control and without control: (**a**) rice plants infected with RTSV; (**b**) rice plants infected with RTBV; (**c**) rice plants infected with RTSV+RTBV; (**d**) green leafhopper vector infected with RTSV; (**e**) green leafhopper vector infected with RTBV; and (**f**) green leafhopper vector infected with RTSV+RTBV.

**Fig. 12:**
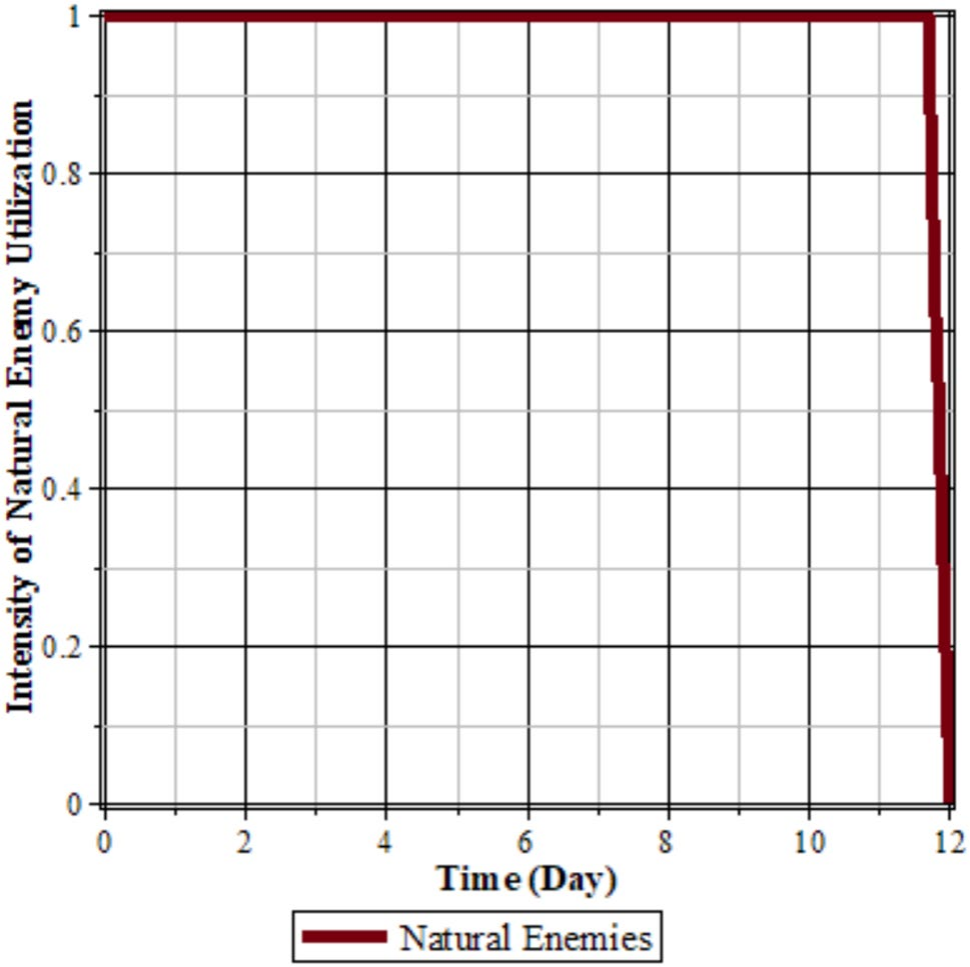
Natural enemies control.

From Figs. 11 to 12, it can be seen that planting refugia plants to attract natural enemies for 12 days can reduce the intensity of the spread of rice tungro disease. This can be seen from the population of rice plants and vectors infected with RTSV, RTBV, and RTSV + RTBV before control in the form of planting refugia plants is more significant when compared to after control in the form of planting refugia plants. In contrast, the population of natural enemies is more critical than when not planting refugia plants. This control dramatically affects the population of infected plants and vectors. This happens because planting refugia plants causes an increase in the population of natural enemies, thereby increasing the possibility of vector death due to the presence of natural enemies.

#### Case 4

**Fig. 13:**
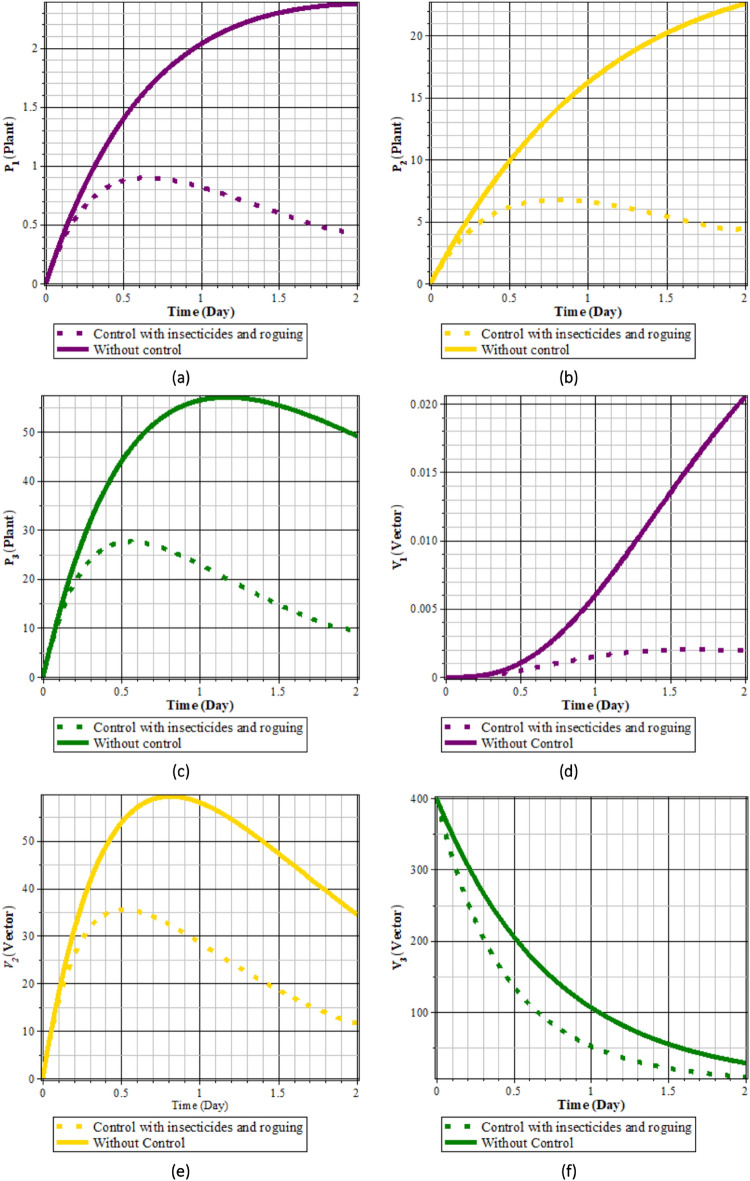
Comparison of populations using insecticides and rouging’ control and without control: (**a**) rice plants infected with RTSV; (**b**) rice plants infected with RTBV; (**c**) rice plants infected with RTSV+RTBV; (**d**) green leafhopper vector infected with RTSV; (**e**) green leafhopper vector infected with RTBV; and (**f**) green leafhopper vector infected with RTSV+RTBV.

**Fig. 14:**
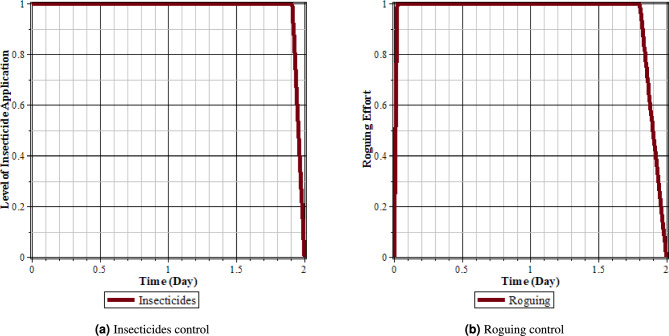
Insecticides and roguing control.

From Figs. 13, 14a and b, it can be seen that roguing and applying insecticides for 2 days can reduce the intensity of the spread of rice tungro disease. This can be seen from the population of rice plants and vectors infected with RTSV, RTBV, and RTSV + RTBV before control in the form of roguing and applying insecticides is more significant when compared to after roguing and applying insecticides. This control dramatically affects infected plants and vectors because by roguing, infected plants will be reduced, impacting the vector population. In addition, control in the form of insecticides reduces the vector population when vectors take food from infected plants that have been applied insecticides.

#### Case 5

**Fig. 15:**
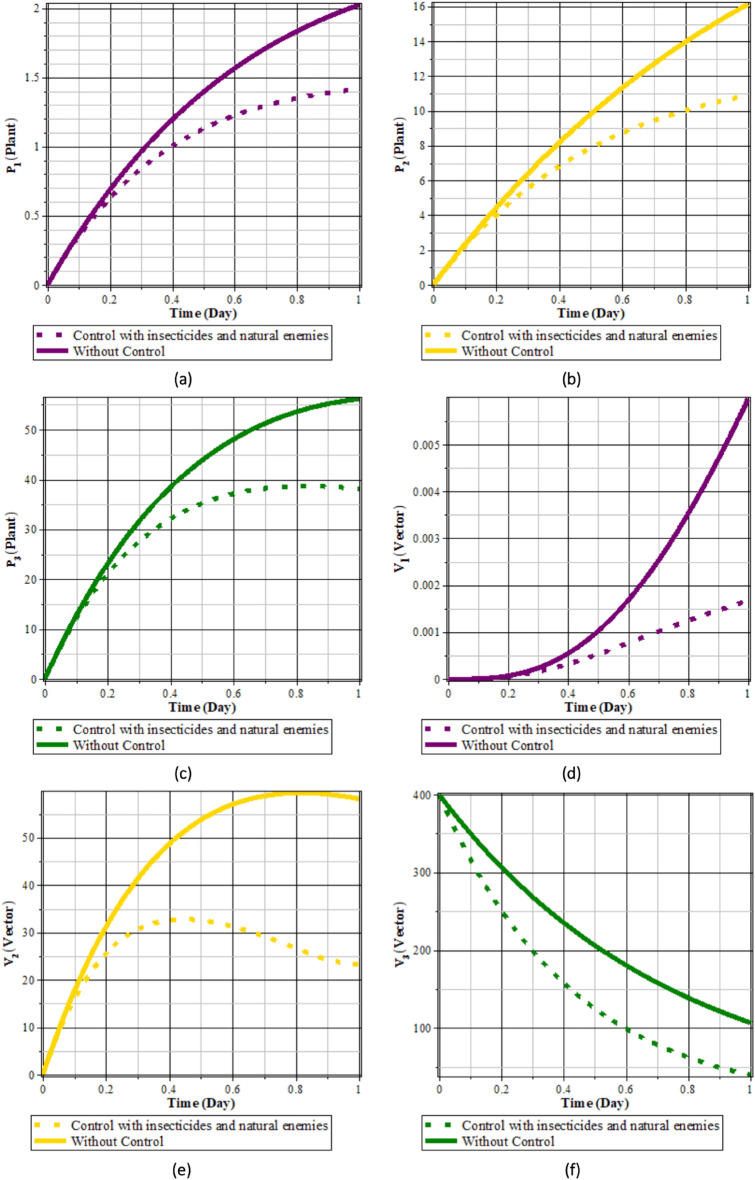
Comparison of populations using insecticides and natural enemies’ control and without control: (**a**) rice plants infected with RTSV; (**b**) rice plants infected with RTBV; (**c**) rice plants infected with RTSV+RTBV; (**d**) green leafhopper vector infected with RTSV; (**e**) green leafhopper vector infected with RTBV; and (**f**) green leafhopper vector infected with RTSV+RTBV.

**Fig. 16:**
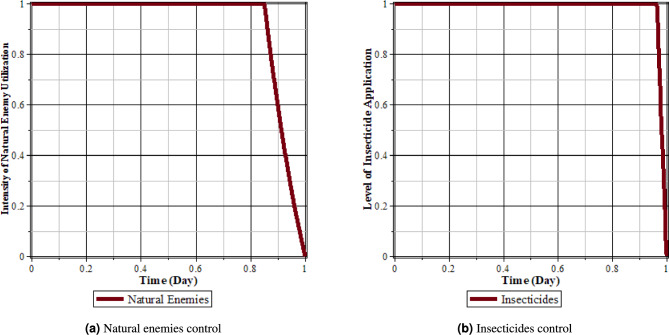
Insecticides and natural enemies control.

From Figs. 15, 16a and b, it can be seen that the intensity of the spread of rice tungro disease can be reduced by carrying out control in the form of natural enemies and insecticides. This can be seen from the population of rice plants and vectors infected with RTSV, RTBV, and RTSV + RTBV before control in the form of natural enemies and insecticides was more significant than after control in the form of natural enemies and insecticides. This control dramatically affects the population of infected plants and vectors. This happens because when planting refugia plants, the population of natural enemies increases, thereby increasing the possibility of vector death due to natural enemies. The application of insecticides to infected plants causes the rate of vector death to increase when taking food from infected rice plants.

#### Case 6

**Fig. 17:**
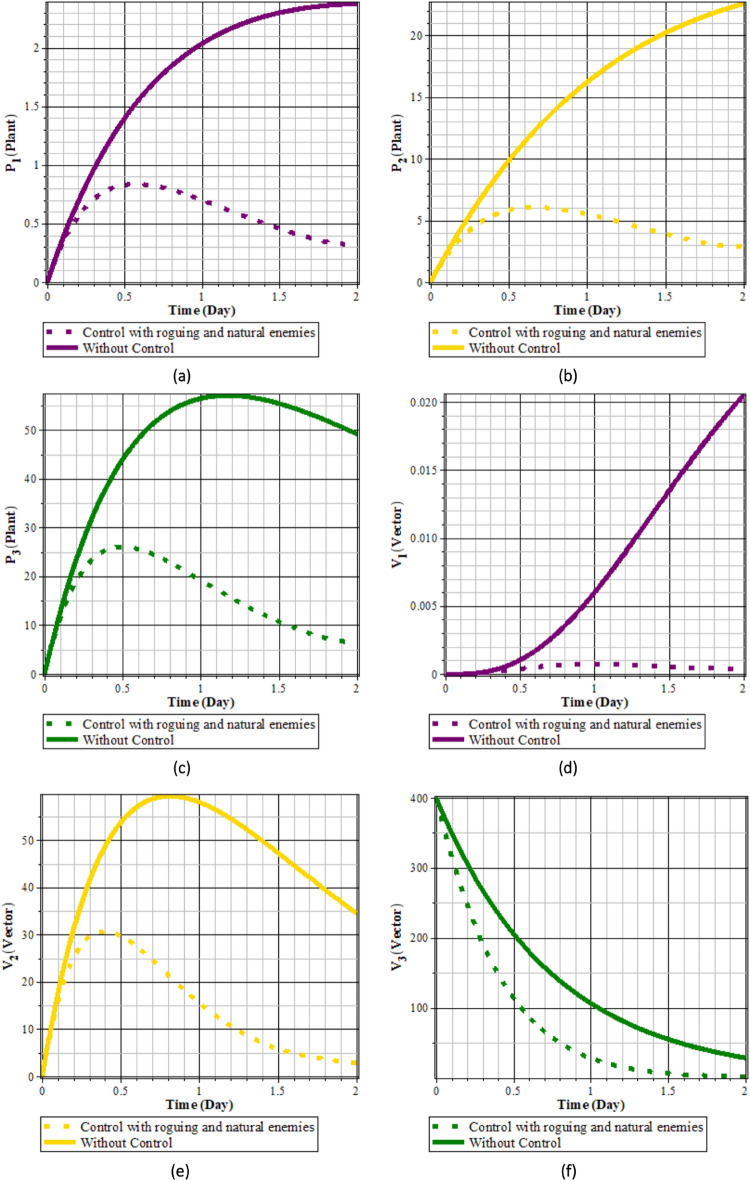
Comparison of populations using roguing and natural enemies’ control and without control: (**a**) rice plants infected with RTSV; (**b**) rice plants infected with RTBV; (**c**) rice plants infected with RTSV+RTBV; (**d**) green leafhopper vector infected with RTSV; (**e**) green leafhopper vector infected with RTBV; and (**f**) green leafhopper vector infected with RTSV+RTBV.

**Fig. 18:**
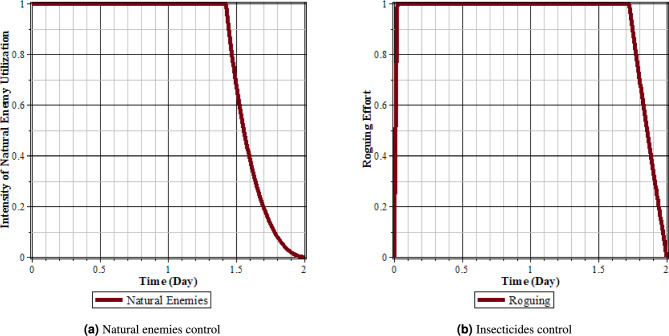
Natural enemies and Insecticides control.

From Figs. 17, 18a, and b, it can be seen that by carrying out control in the form of roguing and natural enemies, the intensity of the spread of rice tungro disease can be reduced. This can be seen from the population of rice plants and vectors infected with RTSV, RTBV, and RTSV + RTBV before control in the form of roguing and natural enemies was more significant when compared to after control in the form of roguing and natural enemies. This control dramatically affects the population of infected plants and vectors. This happens because when planting refugia plants, the population of natural enemies increases, thereby increasing the possibility of vector death due to natural enemies. In addition, control in the form of roguing dramatically affects the population of infected plants and vectors because carrying out roguing will reduce infected plants, which will impact the vector population.

#### Case 7

**Fig. 19:**
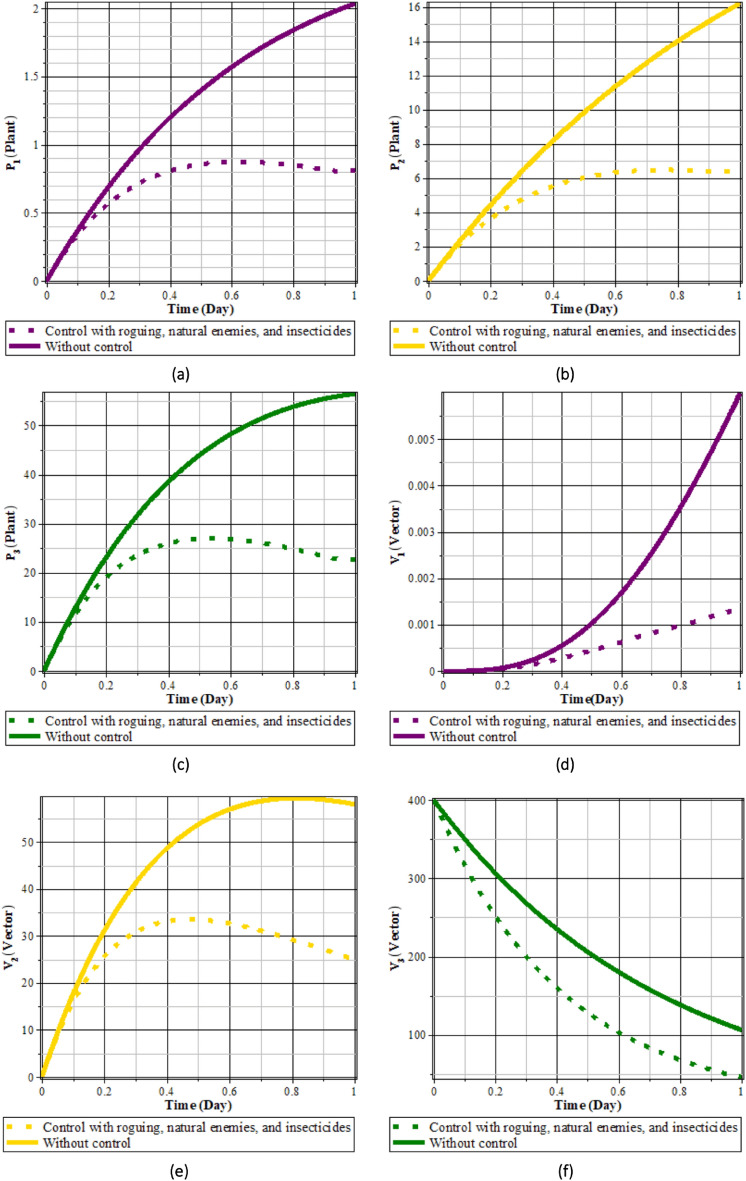
Comparison of populations using roguing, natural enemies and insecticides’ control and without control: (**a**) rice plants infected with RTSV; (**b**) rice plants infected with RTBV; (**c**) rice plants infected with RTSV+RTBV; (**d**) green leafhopper vector infected with RTSV; (**e**) green leafhopper vector infected with RTBV; and (**f**) green leafhopper vector infected with RTSV+RTBV.

**Fig. 20:**
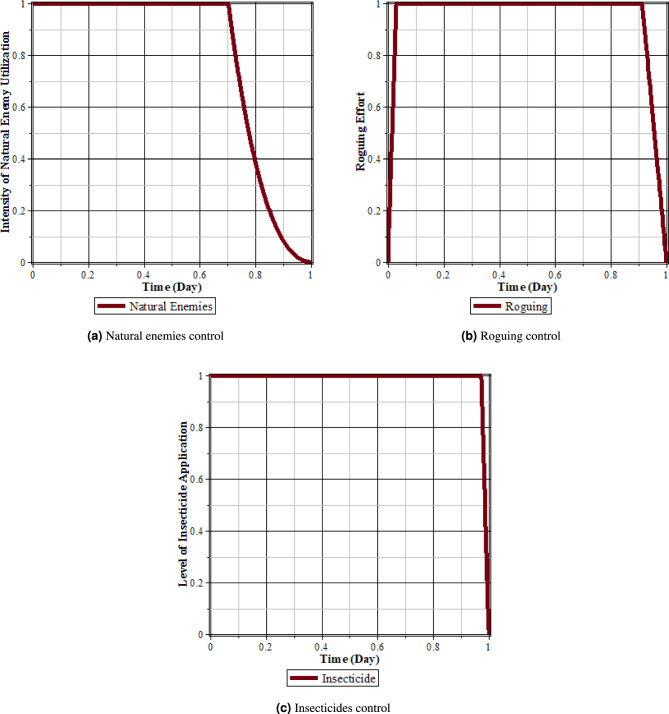
Natural enemies, roguing, and insecticides control.

From Figs. 19, 20a, b, and c, it can be seen that carrying out control in the form of roguing, insecticides, and natural enemies can reduce the intensity of the spread of rice tungro disease. This can be seen from the population of rice plants and vectors infected with RTSV, RTBV, and RTSV + RTBV before control in the form of roguing and natural enemies is more significant when compared to after control in the form of roguing and natural enemies. This control dramatically affects the population of infected plants and vectors. This happens because when planting refugia plants, the population of natural enemies increases, thereby increasing the possibility of vector death due to natural enemies. In addition, control in the form of roguing dramatically affects the population of infected plants and vectors because carrying out roguing will reduce infected plants, which will impact the vector population.

## Conclusion

The mathematical model for the spread of rice tungro disease–considering control through roguing, the planting of refugia plants to conserve natural enemies, and the application of insecticides–consists of nine compartments. Four compartments represent the rice plant population, four represent the green leafhopper (vector) population, and one compartment accounts for the natural enemies.

The analytical and numerical results indicate that the disease-free equilibrium is locally asymptotically stable when the basic reproduction number satisfies $R_0 < 1$, and the endemic equilibrium becomes stable when $R_0> 1$. This behavior is illustrated in the population dynamics plots, where for $R_0> 1$, the infected compartments persist over time, while for $R_0 < 1$, they decline toward zero.

Sensitivity analysis demonstrates that all three control strategies–roguing, insecticide application, and natural enemy conservation–effectively reduce disease prevalence. Among these, the combined application of chemical control (insecticides) and biological control (natural enemies) appears to be the most efficient strategy in reducing infected populations.

However, this conclusion is not solely based on whether the infected populations reach zero, but also considers the rate of decline, the time required to reach near-zero infection, and the cost implications embedded in the objective function. The comparative analysis across the seven control scenarios (individual and paired strategies) reveals differences in effectiveness. While all control cases result in decreased infection levels, some strategies lead to faster initial decline, whereas others may show slower but more sustained reductions. For example, combined control strategies generally result in a more rapid suppression of the infected vector and plant populations than single interventions, even if the time to reach eradication may vary.

Therefore, the recommendation for combining insecticide application and natural enemy conservation is based on a balance between speed of reduction, cost efficiency, and overall impact on disease dynamics, as observed from the simulation graphs and the optimal control outcomes.

## Data Availability

All data generated or analyzed during this study are included in this published article.
